# Thermodynamic and sequential characteristics of phase separation and droplet formation for an intrinsically disordered region/protein ensemble

**DOI:** 10.1371/journal.pcbi.1008672

**Published:** 2021-03-08

**Authors:** Wen-Ting Chu, Jin Wang

**Affiliations:** 1 State Key Laboratory of Electroanalytical Chemistry, Changchun Institute of Applied Chemistry, Chinese Academy of Sciences, Changchun, Jilin, People’s Republic of China; 2 Department of Chemistry & Physics, State University of New York at Stony Brook, Stony Brook, New York, United States of America; University of Maryland School of Pharmacy, UNITED STATES

## Abstract

Liquid–liquid phase separation (LLPS) of some IDPs/IDRs can lead to the formation of the membraneless organelles *in vitro* and *in vivo*, which are essential for many biological processes in the cell. Here we select three different IDR segments of chaperon Swc5 and develop a polymeric slab model at the residue-level. By performing the molecular dynamics simulations, LLPS can be observed at low temperatures even without charge interactions and disappear at high temperatures. Both the sequence length and the charge pattern of the Swc5 segments can influence the critical temperature of LLPS. The results suggest that the effects of the electrostatic interactions on the LLPS behaviors can change significantly with the ratios and distributions of the charged residues, especially the sequence charge decoration (SCD) values. In addition, three different forms of swc conformation can be distinguished on the phase diagram, which is different from the conventional behavior of the free IDP/IDR. Both the packed form (the condensed-phase) and the dispersed form (the dilute-phase) of swc chains are found to be coexisted when LLPS occurs. They change to the fully-spread form at high temperatures. These findings will be helpful for the investigation of the IDP/IDR ensemble behaviors as well as the fundamental mechanism of the LLPS process in bio-systems.

## Author summary

The membraneless organelles caused by liquid–liquid phase separation (LLPS) of IDPs/IDRs are involved in a wide range of biological functions such as RNA processing, ribosome biogenesis, and sequestration of mRNA, proteins, and compacted chromatin. In this study, we focus on the histone H2A-H2B binding partner, Swc5 and investigate the effects of the temperature, sequence length and number of charged residues on the LLPS behaviors. Moreover, we proposed three forms of swc conformation in ensemble according to the phase diagram. These characteristics of conformational changes (from LLPS to no LLPS, from condensed-phase to dilute-phase) are observed in the swc chains with different sequence length and charge pattern, which may be the general property for the IDP/IDR ensembles.

## Introduction

In living cells, there is one special kind of organelles which are lack of the enclosing membrane. They are named as membraneless organelles (MLOs) [1–3], such as nucleoli, stress granules, P bodies, pericentriolar material and germ granules [3–6]. The MLOs are widely spread within the cell nucleus and the cytoplasm, and involved in a wide range of biological functions including RNA processing, ribosome biogenesis, and sequestration of mRNA (for later translation), proteins (for signaling), and compacted chromatin (for gene silencing) [7]. The recent studies revealed that the MLOs behave like liquid droplets with higher density than the surroundings, which can be observed directly via microscopy [4, 8]. The distinct subcellular compartments were deduced to be formed through a process known as liquid–liquid phase separation (LLPS) [5, 9, 10].

LLPS can be found in the solution of one or more kinds of proteins, proteins and RNAs mixture, proteins with crowders *in vitro* [11]. One class of these proteins is the folded proteins with multivalent interactions, including the signaling protein WASP [8]; the other class is the proteins with intrinsically disordered regions (IDRs), such as FUS [12], TDP-43 [13], hnRNPA1/hnRNPA2 [6, 14], Ddx4 [2], LAF-1 [15], etc. Experimental results suggest that the LLPS is sensitive to the temperature and salt concentration. The droplets can easily dissolve upon raising the temperature or salt concentration, and can reform when conditions are reverted. The reversibility comes because the two phases, condensed-phase (droplet) and dilute-phase (dispersed), are in thermodynamic equilibrium [10, 16]. Moreover, the reversible LLPS can be changed to the irreversible pathological aggregates by mutation, phosphorylation, methylation or ubiquitination on the proteins, forming fibrillization that may be a key reason of the neurodegenerative diseases such as Alzheimer’s, ALS, Parkinson’s, and cataract [6, 12, 17, 18]. As a result, protein sequence is also important for the properties of LLPS.

Intrinsically disordered proteins (IDPs) or IDRs contain much more charged residues than folded proteins/regions, thus are often studied in exploring of the relationship of protein dynamics and function such as in signal transduction and gene regulation [19–22]. However, things may be different for the ensemble/solution of many IDP/IDR molecules. In addition, the experiment has revealed that the distribution of the charged residues in Ddx4 determines its LLPS behavior [2]. Combining the Monte Carlo simulations with the extended Flory-Huggins (FH) theory (random-phase-approximation (RPA) polymer theory) [23–25], Das *et al*. constructed a coarse-grained model for the ensemble of the proteins with designed charge distributions to investigate the dependence of the LLPS on the charge pattern of the protein [26, 27]. However, these chains are all ideal ones with identical numbers of positive-charged residues and negative-charged residues. Then Dignon *et al*. discussed the phase characteristics of FUS, LAF-1, hnRNPA2, TDP-43, as well as their variants by using the *Debye*-*Hückel* model [28, 29] for charge–charge interactions [30, 31].

Swc5 as a chaperon protein, which interacts with the histones dimer H2A-H2B and participates in the eviction step of the exchange between H2A and H2A.Z [32], contains a 147 amino acids IDR (1–147) on its N-terminus. The first 79 amino acids (1–79) with the conserved DEF/Y motifs (DEF/Y-1 and DEF/Y-2) have been found to be essential for the binding with H2A-H2B [33]. The Swc5 is a subunit of SWR essential for its H2A.Z deposition activity *in vivo* and *in vitro* [32–34]. Recently, the crystal structure of the histone binding domain of Swc5 in complex with an H2A-H2B dimer has been resolved [33], which indicates that the consecutive acidic residues and flanking hydrophobic residues of swc5 form a cap over the histones, excluding histone-DNA interaction. It supports a model in which swc5 acts as a wedge to promote H2A-H2B dimer eviction. Huang *et al*. showed that the IDR of Swc5 may recruit or retain histones within phase-separated liquid droplets where the transcription factors and the chromatin remodeling factors coalesce to regulate transcription [33]. The charged-residues on the DEF/Y motifs of Swc5 may play an important role in the binding process and phase behavior of Swc5. However, by far there is lack of investigations on the phase separation of Swc5 or other Swc family members. In order to find out the role of the IDR and the DEF/Y motifs in the LLPS of Swc5, we select the first 79 amino acids of Swc5 IDR (denoted as swc 1–79) and cut it into one part with the DEF/Y motifs (swc 1–32) as well as the other part without the DEF/Y motifs (swc 33–79). This setup is expected to capture the main function of Swc5 based on the more reliable structure information of segments. In this study we will take efforts to explore two important issues via molecular simulations: the temperature, sequence length, and charge pattern dependence of phase separation and droplet formation of swc chain ensemble; the different conformational forms of swc chain ensemble under special phase (trinity form). Consequently, this study will uncover the underlying mechanisms of LLPS in bio-molecules and provide helpful insights into the further research of the role of Swc5 in the assembly of histone/chromatin and DNA replication.

## Materials and methods

### Slab simulation model

**Fig 1:**
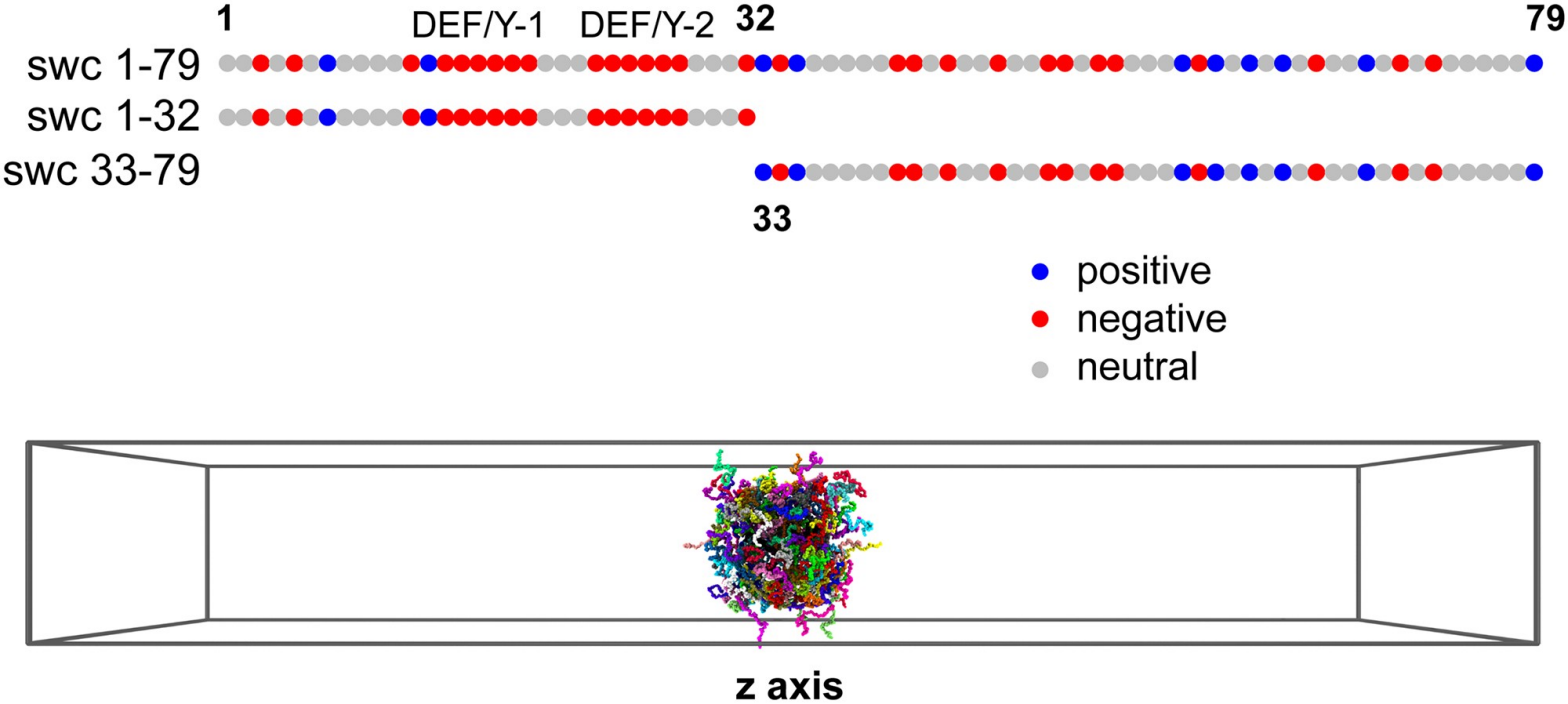
The three swc chains prepared for the simulations (top) and the slab simulation model (bottom). Top: one bead represents one residue of protein. Positive-charged, negative-charged, and neutral residues are shown as blue, red, and gray beads, respectively. The DEF/Y-1 and DEF/Y-2 motifs are labeled on the sequence. Bottom: in the initial state of the long-time simulation, 200 swc chains (*n* = 200, same sequence) were added into the periodic rectangular box (*z* axis = 300 nm).

We developed a coarse-grained model for each swc sequence, representing each residue of the complex by one bead on the C_*α*_ atom. Here in this study we selected three different kinds of Swc5 segments, swc 1–79, swc 33–79, and swc 1–32 (see Fig 1). Therefore, the sequence length (or the bead number of each chain, denote as *L*) is 79, 47, and 32, respectively. Then three different simulation systems were built for these swc chains. In each system, 200 number of identical swc chains (*n* = 200) were added in the simulation box. Therefore the total number of beads (*N* = *n* × *L*) in each system is 15800, 9400, and 6400, respectively. Besides, we tested the Langevin dynamics simulations of two model sequences, sv1 and sv15 (from [35] and [26], 50 amino acids in each sequence), with the same simulation settings.

In this study we introduce a potential energy function of the system, which includes bonded, pairwise interactions (12-6 Lennard-Jones (LJ) potential), as well as the electrostatic interactions (Debye-H*ü*ckel potential). The mean bond length is 3.8 Å (*a* = 3.8 Å). Here in this study, we used the standard Lennard-Jones potential $\varepsilon [{\left(\frac{\sigma }{r}\right)}^{12}-{\left(\frac{\sigma }{r}\right)}^{6}]$. The parameters *σ* and *ε* were carefully calibrated with the single chain of swc 1–79 before the long-time Langevin dynamics simulation of 200 chains system. Swc5 consists of an intrinsically disordered region (IDR) on the N-terminal half of the 303-amino acid polypeptide [33]. Because there are no structures of *apo* Swc5 available (the only crystal structure 6KBB is H2A-H2B-bound swc 15–32), we built the swc 1–79 model according to the properties of common isolated IDP (random coil). Firstly, parameter *σ* is the “finite distance”. *σ*_*ij*_ is the optimal distance between beads *i* and *j* that are in contact with each other. We calculated the non-local distances (e.g. 1–5) of the parts of the swc chains in 6KBB without the secondary structures (coil parts). These distance values are from 8 to 12 Å. Therefore, we set the parameter *σ* to 10 Å (about 2.6 *a*, *a* is the mean bond length). The parameter *ε* is the well depth and the energy responsible for the strength of a structure. Lower *ε* will increase the flexibility of the structure. In addition, we performed a series of test Langevin dynamics simulations on swc 1–79 and found the LJ potential and coulomb potential at the same level when the parameter *ε* equals to 0.01 kJ/mol (*σ* = 1.0 nm). Then the head–end distance (D) show that the isolated swc 1–79 behaves like a flexible IDP when *σ* = 1.0 nm and *ε* = 0.01 kJ/mol (see Supporting information S1 Fig).

The electrostatic interaction is calculated by the *Debye*-*Hückel* model [20, 28, 29, 36, 37], which can be used to quantify the strength of the charge–charge attractions and repulsions in varying ionic strengths:

$$\begin{array}{c} V_{Debye-H\ddot{u}ckel}=K_{coulomb}B\left(\kappa \right)\sum_{i,j}\frac{q_{i}q_{j}\exp(-\kappa r_{ij})}{\epsilon r_{ij}} \end{array}$$ (1)

In Eq 1, *K*_*coulomb*_ = 138.94 *kJ* · *mol*^−1^ · *nm* · *e*^−2^ is the electric conversion factor; *B*(*κ*) is the salt-dependent coefficient; *κ*^−1^ is the Debye screening length, which is directly influenced by the solvent ionic strength (IS)/salt concentration *C*_*salt*_ ($\kappa \approx 3.2\sqrt{C_{salt}}$); *ϵ* is the dielectric constant, which was set to 80 during the simulations to mimic the solvent medium (water). In our model, Lys and Arg have a positive point charge (+ e), Asp and Glu have a negative point charge (−e). By using this model, the strength of the electrostatic interactions can be affected by the salt solvent through the screening effect. In order to investigate the role of electrostatic interactions, two extreme cases were designed for each system. One is 10 mM salt concentration (*C*_*salt*_ = 0.01 M), which represents the system with extremely high strength of electrostatic interactions; the other is infinite high salt concentration, which means that all the electrostatic interactions are screened by the solvent (no charges/electrostatic interactions).

In order to observe the liquid–liquid phase separation (LLPS), determine the phase diagram, and save the computational costs, the slab model was introduced for the simulations [30, 31, 38, 39]. Similar simulation strategy is applied as the previous studies [30, 31]. The slab model has a very long *z* axis (about 8–12 times of the length of *x*/*y* axis), thus the density distributions and the phase transitions can be explored in one dimension. After the initial minimization, each system was equilibrated for 10 ns at 150 K in NPT ensemble, with periodic boundary conditions and 1.0 bar pressure (Parrinello-Rahman barostat) [40]. The system becomes a packed cubic box after the NPT simulation. Then we change the shape of the periodic simulation box by elongating the *z* dimension to 300 nm (*z* = 300 nm). Consider the different sequence length, we set three different lengths of *x*, *y* axes for swc 1–79 (*x* = *y* = 40 nm), swc 33–79 (*x* = *y* = 30 nm), and swc 1–32 (*x* = *y* = 25 nm) to ensure the similar ratio of bead number and volume. Because the sequence length of both sv1 and sv15 is 50, similar to swc 33–79, the simulation box of sv1/sv15 is the same as that of swc 33–79. Then a 5 *μ*s long-time Langevin dynamics run in NVT ensemble was performed on each system, keeping the total protein density constant. All simulations were performed with Gromacs 4.5.5 [41] with 2.0 fs time step and 1.0 ps^−1^ friction coefficient of Langevin thermostat. In this study, we use a simplified coarse-grained with reduced units (*k*_*B*_ *T* = 1). Therefore, the simulation temperature/time is in reduced unit and does not equal to the real temperature/time. The simulation temperature was set from 100 K to 400 K (100, 150, 200, 300, and 400 K) in Gromacs to uncover its effect on LLPS. Here we set the unit temperature (*T*_0_) and unit time (*τ*) to 100 K and 1 ns in Gromacs. As a result, the simulation temperatures correspond to 1.0, 1.5, 2.0, 3.0, and 4.0 *T*_0_ and the simulation length of each trajectory corresponds to 5000 *τ*. Generally, for folded protein, one can estimate the physiological temperature through the ratio of experimental melting temperature and the simulated folding temperature [20, 42]. However, Swc5 is a IDP that does not fold at room temperature. As a result, the model used in this study was developed for IDP chains. The simulation temperature cannot be transformed to the real temperature directly. But the trends of simulated *T*_*cr*_ values can reveal the probability of LLPS behaviors.

### Data analysis

To determine the phase distribution of the swc system, for each frame of the trajectory we select the *z* axis as the reaction coordinate and cut it into 30 windows (*γ*, the length of *γ* is 10 nm). Then we count the number of the beads in each window (denote as *m*_*γ*_). For each swc system has different total number of beads (*N*), here we calculate the distribution of the fraction of beads ($P_{\gamma }=\frac{m_{\gamma }}{N}$, *γ* = 1..30) and find out the highest and the lowest values of this distribution (*P*_*H*_ and *P*_*L*_). If LLPS occurs, the condensed-phase and dilute-phase are coexisted in the system. As a result, there is a large difference between *P*_*H*_ and *P*_*L*_. In addition, it should be noted that at the same *P*_*γ*_, the density of swc 1–79 is much higher than that of swc 33–79 and swc 1–32 because of the large total number of beads (*N*). As LLPS disappears, the system becomes uniform and the difference between *P*_*H*_ and *P*_*L*_ gets smaller. However, we can not obtain identical *P*_*H*_ and *P*_*L*_ because of the limits of the sampling. Therefore, the *P*_*H*_ and *P*_*L*_ values will get close at high simulation temperature.

For each frame of the simulation trajectory, the head-end distance (*D*) was calculated for the first and the last bead of each chain (e.g. distance between beads 1 and 79 for swc 1–79). In order to quantify the strength of the electrostatic interactions of the swc chains with and without LLPS, we counted the number of intra-chain electrostatic attractions (*E*_*intra*_) of each chain and inter-chain electrostatic attractions (*E*_*inter*_) of each chain–chain pair. In detail, if the distance between two bead *i* and *j* with opposite charges is lower than the cut-off distance (1.2 nm, 1.2 times of *σ* in LJ potential), we denote that there is one electrostatic attraction between the two beads. If *i* and *j* belong to the same chain, this interaction is collected as the intra-chain electrostatic attraction; if *i* and *j* belong to different chains, this interaction is collected as the inter-chain electrostatic attraction. In addition, here we only calculated the electrostatic interactions of the system at 10 mM salt concentrations since all the electrostatic interactions are screened by the solvent in the other case (no charge). Therefore, 200 *D*, *E*_*intra*_, and *E*_*inter*_ values can be collected for each frame of the simulation trajectory.

In order to show the distribution of all the 200 chains, we set the center of the system (condensed-phase with LLPS) at the zero point of *x*, *y*, and *z* axes. All the chains were located on both negative and positive sides of the zero point. Then we calculated the distance/displacement of each chain (center of mass (CM)) to the zero point on the *z* axis (|*z*|_*n*_ = |*z*_*n*_ − *z*_0_|, n = 1..200), which can reveal the distribution of the 200 chains from the center of the system. Since the length of the simulation box on the *z* axis is 300 nm, the largest |*z*| is 150 nm. We then selected the |*z*| as the reaction coordinate and cut it into 30 windows. The fraction of the number of the chains that belong to each window, the standard error of the |*z*| values in each window, as well as the distribution of *D*, *E*_*intra*_, and *E*_*inter*_ along the reaction coordinate.

Here in this study we also calculated the radial distribution functions (rdf). The rdf (*g*(*r*)) defines the probability of finding a group of atoms at a distance *r* from another group of atoms. The *r* value corresponds to the peak value of *g*(*r*) curve shows the distance between the two groups with the highest probability. As a result, we simply calculated the *g*(*r*) curves between chain 1 and another chain (chain 2, 3, …, 200) and collected the *r* values corresponding to the peak value of *g*(*r*) curves (peak1).

## Results

### Temperature, sequence length, and charge pattern affect the LLPS of swc

Three different kinds of swc chains were selected for the simulations of the assembling and diffusing processes at different conditions. As shown in Fig 1, first 79 amino acids of swc (denote as swc 1–79) is rich in charged amino acids, including 29 negative-charged amino acids and 10 positive-charged amino acids. The front 32 amino acids form the shorter part of swc 1–79, swc 1–32, which includes the conserved DEF/Y-1 and DEF/Y-2 motifs. Swc 1–32 has only 2 positive-charged amino acids, but many negative-charged amino acids (16). The back-end 47 amino acids were truncated into the longer part of swc 1–79, swc 33–79, which has 13 negative-charged amino acids and 8 positive-charged amino acids.

We use one bead to represent one residue of proteins. To observe the phase behaviors of different swc chains, a model with many identical swc chains was built for the molecular simulation. For each simulation, 200 chains (*n* = 200) of swc (swc 1–32 or 33–79 or 1–79) were added into the periodic rectangular box to build the slab simulation model (see Fig 1), which has short *x* and *y* axes but an elongated *z* axis [30, 31]. The simulation boxes of swc 1–32, swc 33–79, swc 1–79 has the same length of *z* axis (300 nm, about 750 a), but different lengths of *x* and *y* axes (*x* = *y* = 25, 30, and 40 nm). Each protein can be considered as a polymer, which randomly walks in the simulation box but show regular behaviors under special conditions.

**Fig 2:**
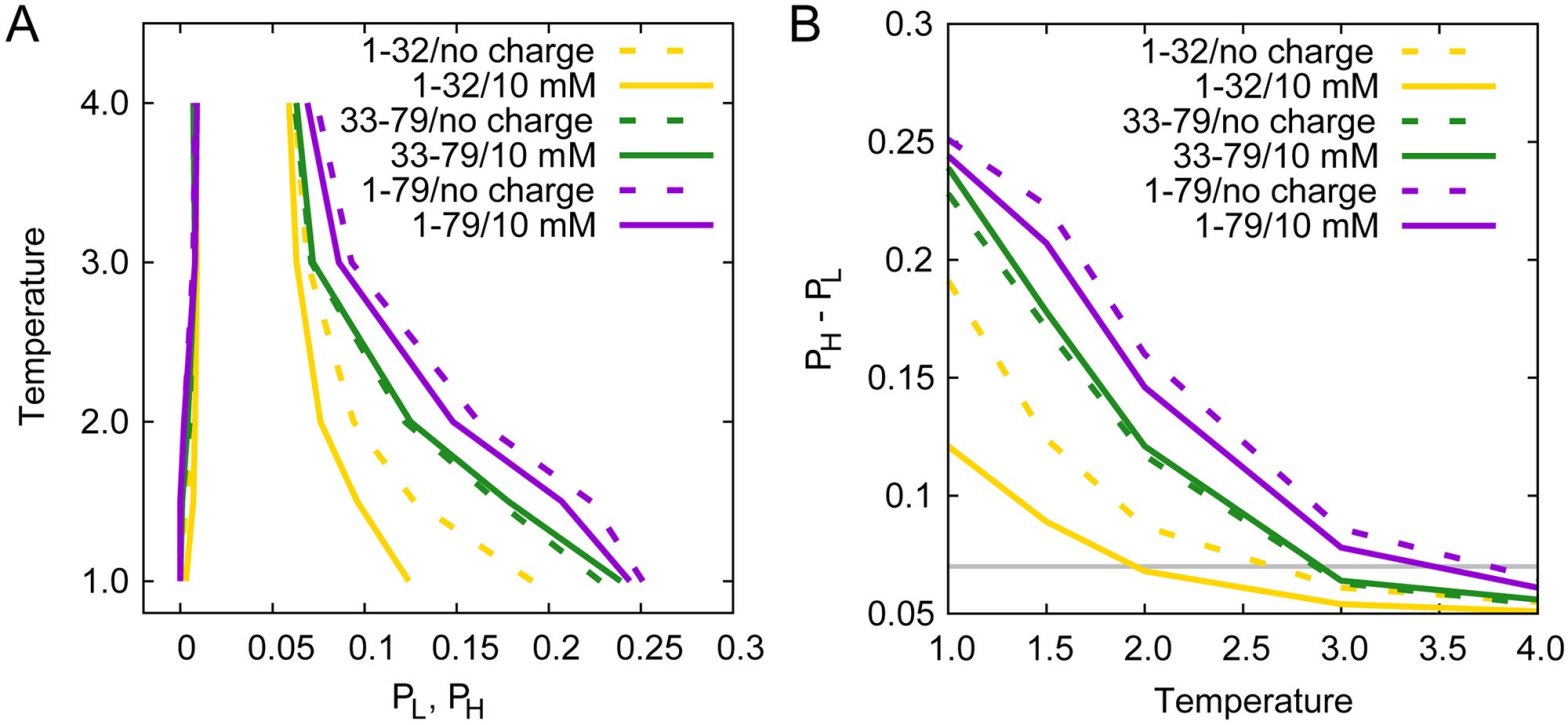
The appearance and disappearance of phase separation. (A) Phase diagram of swc chains in different solvents. (B) *P*_*H*_ − *P*_*L*_ changes with temperature and solvent. Here *P*_*H*_ and *P*_*L*_ are the highest and the lowest points of swc residue distribution along the *z* axis. The *P*_*H*_ and *P*_*L*_ values are calculated with the last 1000 *τ* simulation data, as *P*_*H*_ − *P*_*L*_ of all the simulations reaches equilibrium after 3000 *τ* (see Supporting information S5–S7 Figs). The *P*_*H*_ − *P*_*L*_ data are listed in Supporting information S1 Table.

Simulations were performed under different temperatures from 1.0 to 4.0 *T*_0_ (1.0, 1.5, 2.0, 3.0, and 4.0 *T*_0_, in reduced units) and different salt concentrations of solvent (10 mM and extremely high salt concentration, corresponding to high and no strengths of electrostatic interactions in the system, denoted as 10 mM and no charge below), respectively. After a long-time equilibration of 5000 *τ* simulation, we can observe LLPS of swc chains at low temperatures in varying degrees. The analysis of the radial distribution function curves (*g*(*r*)) can show the general distribution of chain–chain distance. it is obvious that the chain–chain distances of the system with LLPS (low temperature) tend to be lower than that without LLPS (high temperature), see Supporting information S2–S4 Figs. Here we calculate the distribution of the fraction of the swc residues ($P_{\gamma }=\frac{m_{\gamma }}{N}$, *γ* = 1..30, see the Materials and methods section) along the long *z* axis, as well as the highest and the lowest probability of this distribution (*P*_*H*_ and *P*_*L*_). If there is large difference between *P*_*H*_ and *P*_*L*_, LLPS occurred in the system. As shown in Fig 2, the simulation results suggest the obvious appearance of LLPS for swc 1–79 and swc 33–79 at the temperatures below 1.5 *T*_0_ (*P*_*H*_ − *P*_*L*_>0.15). LLPS can be found for swc 1–32 at 1.0 *T*_0_ if there are no charge interactions in the system (no charge). However, charge interactions of swc 1–32 can have a large effect on its distribution, leading to no obvious LLPS at 1.0 and 1.5 *T*_0_ of swc 1–32 at 10 mM salt concentration.

**Fig 3:**
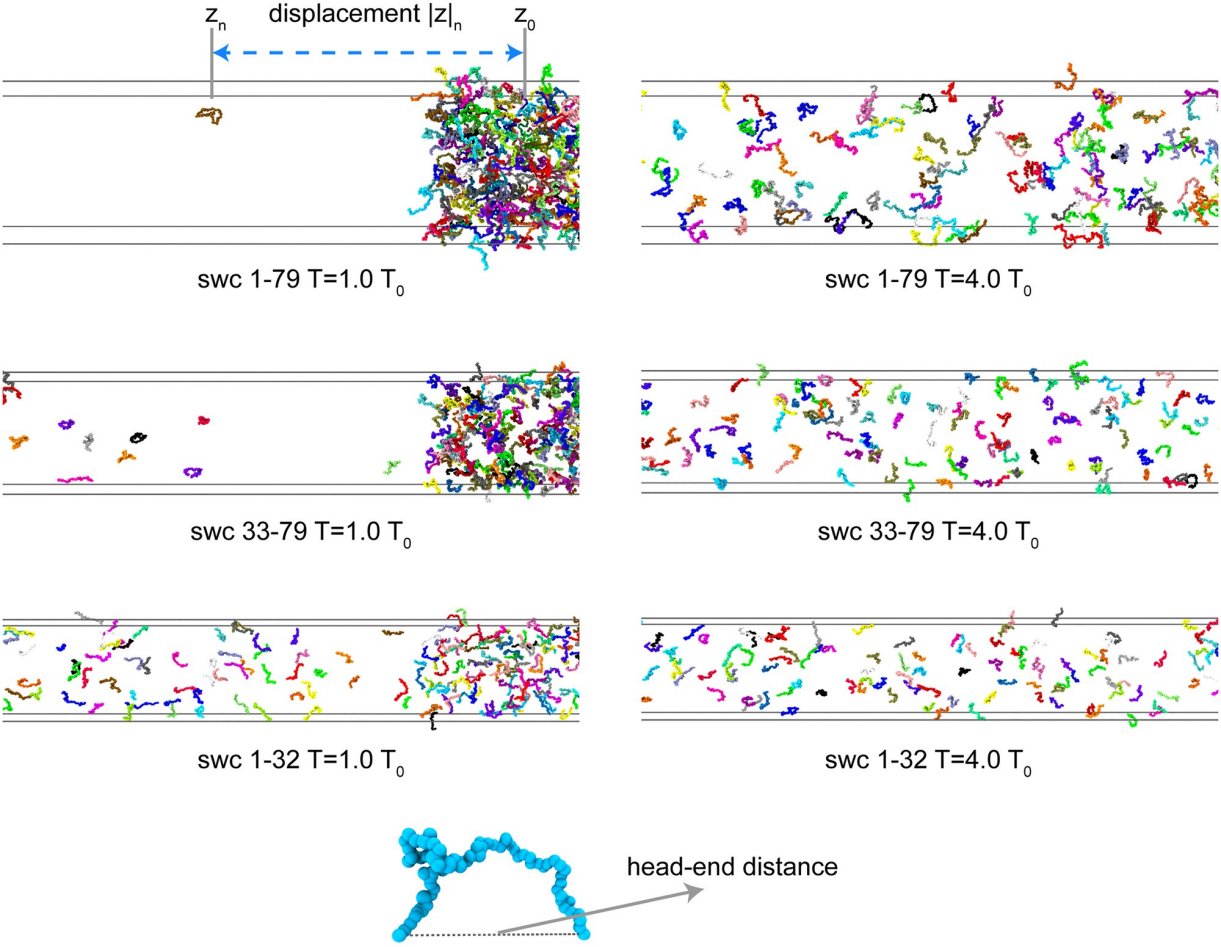
The last frame of the simulations of the swc systems at 1.0 and 4.0 *T*_0_. Here we illustrate part of the long *z* axis. The head–end distance (*D*) and the displacement |*z*| are labeled in this figure. Only the simulations with 10 mM salt concentration are shown in this figure.

We should notice that *P*_*γ*_ represents the fraction of all the polymer beads/protein residues. The total number of beads of swc 1–79 (*N* = 15800) is much higher than that of swc 33–79 (*N* = 9400) and swc 1–32 (*N* = 6400). Therefore, it seems that there are more beads in each window *γ* of swc 1–79 than that of swc 33–79 and swc 1–32. As shown in Fig 3, at 1.0 *T*_0_, LLPS is obvious in the system swc 1–79 and swc 33–79, but not obvious in the system 1–32. However, in order to keep the same total density, the three systems have the same length of *z* axis but different lengths of *x* and *y* axes. As a result, the distribution of density on *z* axis (density in each window: $D_{\gamma }=\frac{m_{\gamma }}{V_{\gamma }}$, *V*_*γ*_ is the volume of each window) has the same trends with the distribution of *P*_*γ*_ among these systems.

The LLPS will disappear at high temperature (critical temperature, *T*_*cr*_) when the density difference is not found in the protein solution. The *T*_*cr*_ can be obtained by fitting to *ρ*_*H*_ − *ρ*_*L*_ = *A*(*T*_*cr*_ − *T*)^*β*^ [30, 31] or the Flory-Huggins theory [23, 24, 43]. Here we use *P*_*H*_ − *P*_*L*_ to roughly estimate *T*_*cr*_ because of the different sequence length of the three swc chains. When *P*_*H*_ − *P*_*L*_ = 0, the temperature is equal to *T*_*cr*_. However, we can not obtain identical *P*_*H*_ and *P*_*L*_ at high temperature in the simulations because of the limits of the sampling. If we set the threshold of LLPS to be 0.07 (LLPS disappears when *P*_*H*_ − *P*_*L*_<0.07), the *T*_*cr*_ values of swc 1–79 and swc 33–79 are about 3.5 *T*_0_ and 3.0 *T*_0_, respectively (see Fig 2B). The salt concentration of the solvent has a significant impact on the *T*_*cr*_ of swc 1–32. The charge interactions will decrease the *T*_*cr*_ of swc 1–32 for about 0.5 *T*_0_ at 10 mM and swc 1–79 for about 0.2 *T*_0_ at 10 mM, and enhance the *T*_*cr*_ a bit of swc 33–79. The changes of *T*_*cr*_ on charge interactions seem to have something to do with the sequence charge decoration (SCD) value ($SCD=\frac{1}{N}\sum_{i=1}^{N}\sum_{j=i+1}^{N}\sigma_{i}\sigma_{j}\sqrt{j-i}$, see Ref [44]). SCD with large absolute value means the sequence with charge-blocky pattern (like sv15 in Ref [44]); SCD with small absolute value means the sequence with charge-scrambled pattern (like sv1 in Ref [44]). The SCD values of swc 1–32, swc 33–79, and swc 1–79 are 7.77, -0.32, and 7.40. The SCD can be considered as 0.00 when there are no charge interactions (neutral polymers). Recently, Das *et al*. discussed the influence of charge distribution on the behaviors and phase diagrams of two sequences sv1 and sv15 (with the same length and total charge number) [26]. We have performed the simulations using the sv1 and sv15 sequences and the obtained same result with Das *et al*. that the *T*_*cr*_ of sv15 is higher than that of sv1 (see the Discussion section). However, in our study the systems have different lengths, total charge numbers, and numbers of the charged residues. Then we compare swc 1–79 with swc 1–32, which have similar SCD values. The results suggest that the charge interactions have a more significant effect on the sequences with shorter length and fewer numbers of the charged residues.

**Fig 4:**
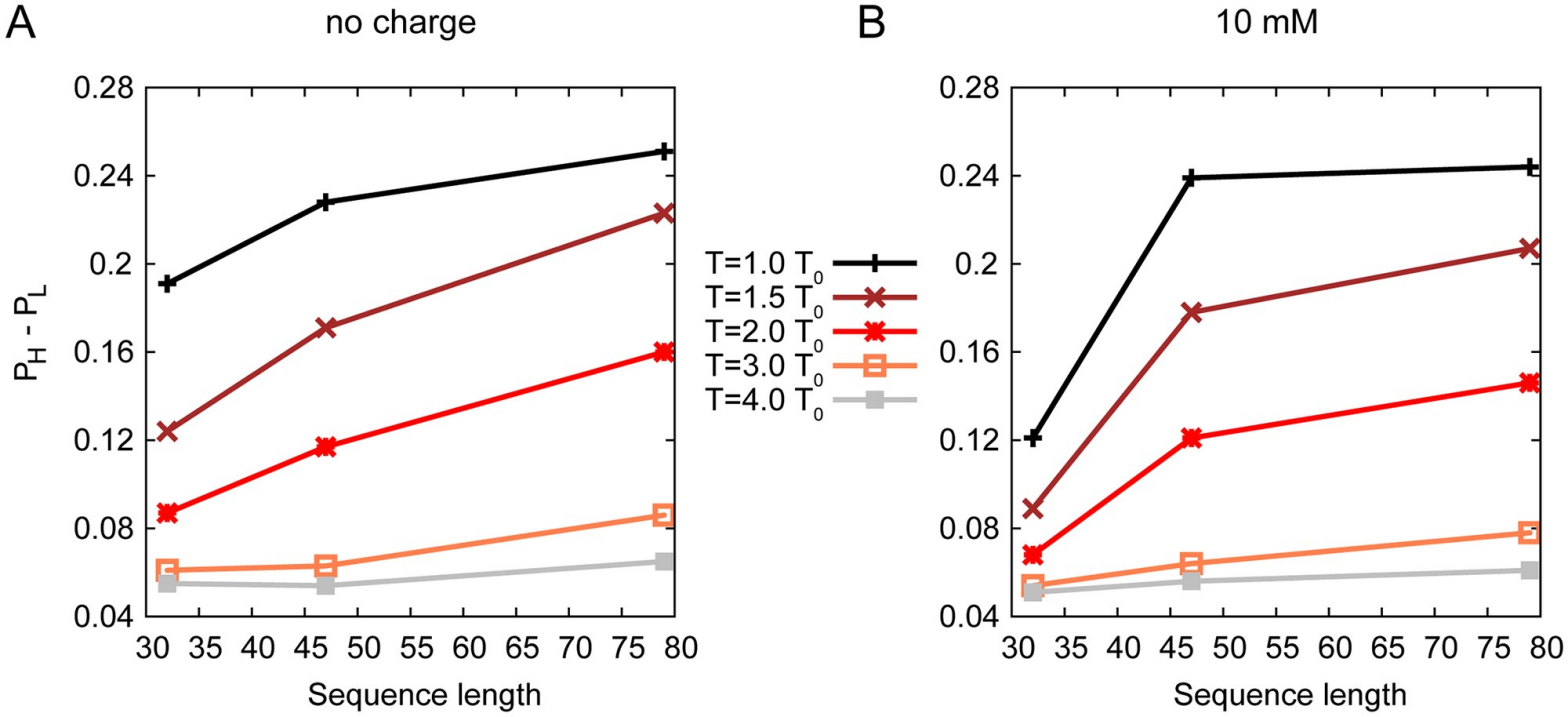
*P*_*H*_ − *P*_*L*_ as a function of swc sequence length. 32 on *x* axis corresponds to swc 1–32, 47 corresponds to swc 33–79, 79 corresponds to swc 1–79.

Moreover, here swc without charge interactions can be considered as neutral polymers, the behaviors of which depend mainly on the length of the polymer chain. Therefore we present a figure to show the influence of the sequence length on the *P*_*H*_ − *P*_*L*_. As shown in Fig 4A, without charge interactions, the extent of LLPS (measured by *P*_*H*_ − *P*_*L*_) and the length of swc chain are linearly correlated. Long polymers prefer to show the LLPS at the same temperature. However, the charge interactions decrease the *P*_*H*_ − *P*_*L*_ of swc 1–32 and 1–79, but increase the *P*_*H*_ − *P*_*L*_ of swc 33–79 (see Fig 4B). At temperature 1.0 *T*_0_, two sequences with different lengths (swc 1–79 and swc 33–79) have similar extent of LLPS under the influence of the charge interactions at 10 mM. Consequently, the distribution of the charged residues will affect the extent of LLPS.

### The droplet is dynamical even at low temperatures

In order to analyze the behavior of the individual chain during the simulation, we calculated the displacement |*z*| of each chain on the *z* axis (|*z*|_*n*_ = |*z*_*n*_ − *z*_0_|, n = 1..200, see Fig 3), which can reveal the movement of each chain relative to the center of the system (condensed-phase with LLPS) during the simulation. As shown in Supporting information S8–S10 Figs, at low temperatures, the composition of the condensed-phase or the dilute-phase is not invariant after equilibration, in spite of the large and stable *P*_*H*_ − *P*_*L*_. Some swc chains move from the condensed-phase to the dilute-phase, or from the dilute-phase to the condensed-phase. This is consistent with the observation of the dynamical LLPS in the experiments [6, 43]. This kind of movement changes to be frequent in the simulation time at high temperatures, suggesting the lost of the LLPS behavior.

We then selected the |*z*| as the reaction coordinate and cut it into 30 windows and calculated the fraction of the number of the chains that belong to each window, as well as the standard error of the |*z*| values in each window. As shown in the Supporting information S11 Fig, high value of the probability of low |*z*| corresponds to the condensed-phase, which is not obvious at high temperature. Intriguingly, at 1.0 *T*_0_, the region of the condensed-phase of swc 33–79 is larger than that of swc 1–79 and swc 1–32. In addition, we used the standard error of the |*z*| values in each window to show the fluctuation. The fluctuation of the chains in dilute-phase is much higher than those in the condensed-phase (see Supporting information S11 Fig). Moreover, at 1.0 *T*_0_, the fluctuation of the condensed-phase of swc 1–79 is a bit lower than that of swc 33–79 and swc 1–32, which means that the condensed-phase of swc 1–79 is more stable than that of the other two sequences.

### Temperature, sequence length, and charge pattern play different roles on the conformation of swc

**Fig 5:**
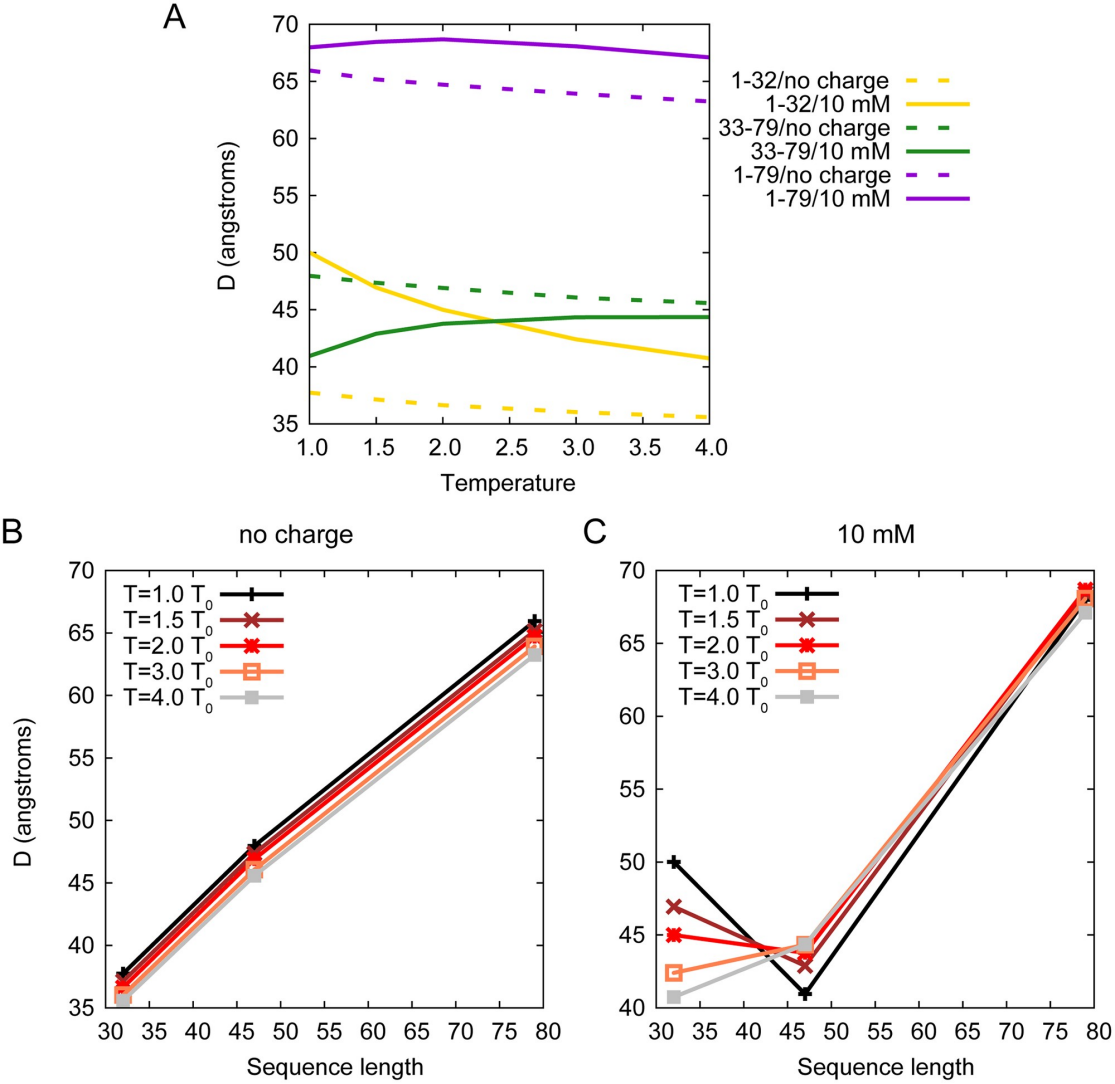
Head–end distance (*D*) distributions. (A) Head–end distance (*D*) of swc chains as a function of temperature. (B–C) Head–end distance (*D*) of swc chains as a function of sequence length during simulations without charges (B) and at 10 mM (C). In panel B and C, 32 on *x* axis corresponds to swc 1–32, 47 corresponds to swc 33–79, 79 corresponds to swc 1–79. The *D* value is calculated as the mean value of the 200 chains in the system during the last 1000 *τ* simulation data.

For each swc chain, we record its head–end distance (see Fig 3, denoted as *D*) to show the conformational changes in the system during the simulation time. As shown in Fig 5A and 5B, for neutral polymers (without charge interactions), the head–end distance (*D*) and the length of swc chain (*L*) are linearly correlated. Compared with the sequence length *L*, the temperature influences the *D* value slightly. Swc chains tend to be a bit more expanded at lower temperatures when there are no charge interactions. The strange behavior observed for the swc ensemble is different from that of the free IDPs which unfold (expanded) at high temperatures. We will explain it in detail in the next section. In addition, the charge interactions of swc 33–79 and sv15 can significantly decrease the *D* value (see Fig 5A and 5C, Supporting information S2 Table). The contribution of the electrostatic attractions is higher than that of the repulsions in the swc 33–79 and sv15 system, making it to be more “folded/collapsed” like at lower temperature. The charge interactions of swc 1–32 and 1–79 have the opposite effect on the *D* value. Especially for swc 1–32, the repulsions straighten the protein significantly at low temperatures. Swc 1–32 have 16 negative-charged and 2 positive-charged residues. As illustrated in Fig 1, the tandem DEF/Y-1 and DEF/Y-2 motifs compose of the continuous negative-charged residues, which will enhance the repulsions significantly at low salt concentrations.

### The ensemble of chains suggest the three forms of swc

**Fig 6:**
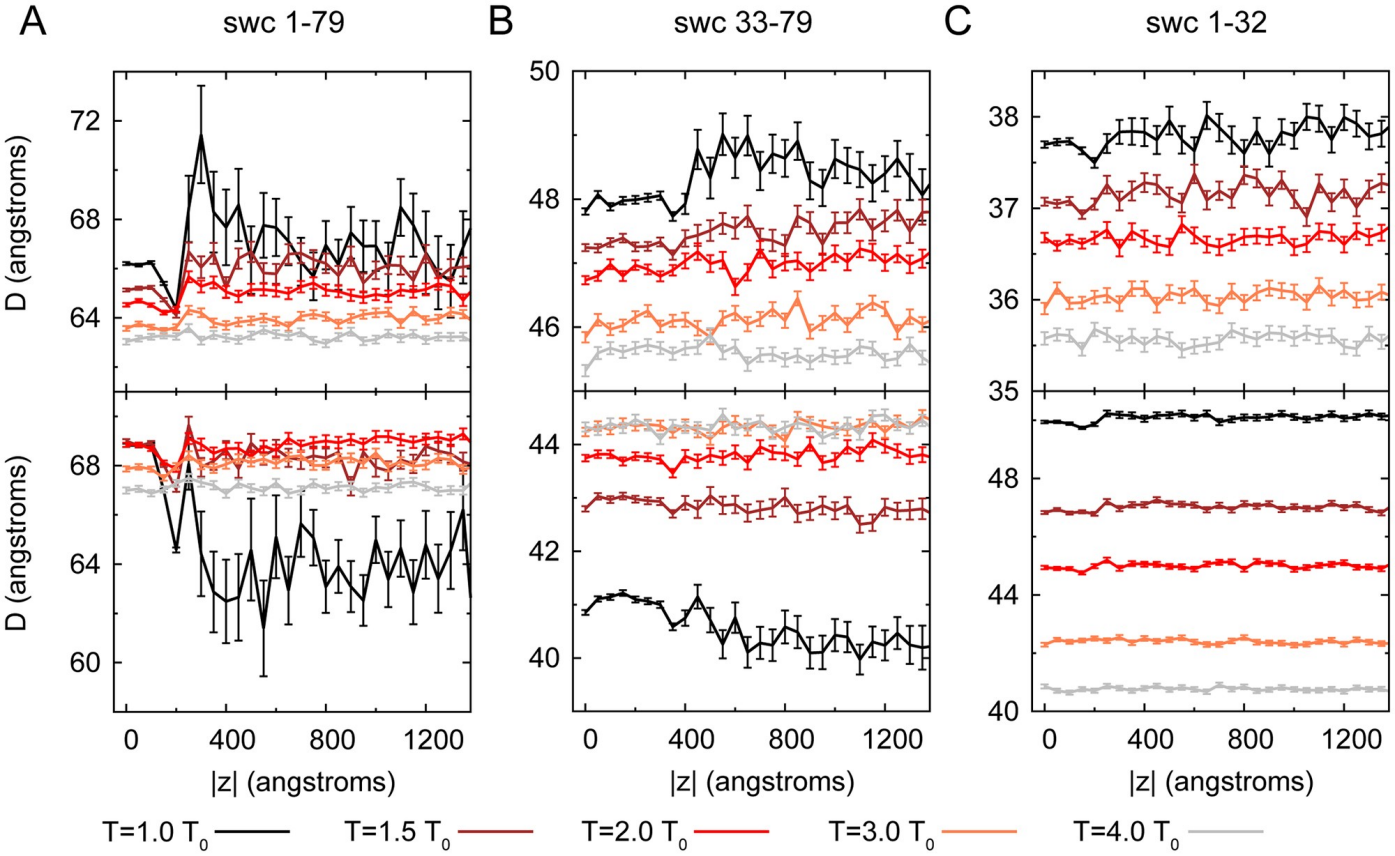
Head–end distance (*D*) of swc 1–79 (A), swc 33–79 (B), and swc 1–32 (C) as a function of displacement z. Data without charges and at 10 mM are shown in the up panels and the bottom panels, respectively. Mean *D* and standard error (*σ*) values during the last 1000 *τ* simulation data are illustrated in this figure. Considering the effect of boundary, the data with displacement z higher than 1400 are not calculated.

Aiming to find out the difference between the chains in the condensed-phase and that in the dilute-phase, we calculated the distributions of the swc head–end distance (referred as *D*) along the displacement |*z*|. In the meantime, the standard error of *D* was collected for each window along the |*z*|, denoted as *σ* (see Fig 6). The large and small *D* values correspond to the extended and collapsed structure of IDPs. In addition, the *σ* value shows the flexibility of IDP structure.

**Fig 7:**
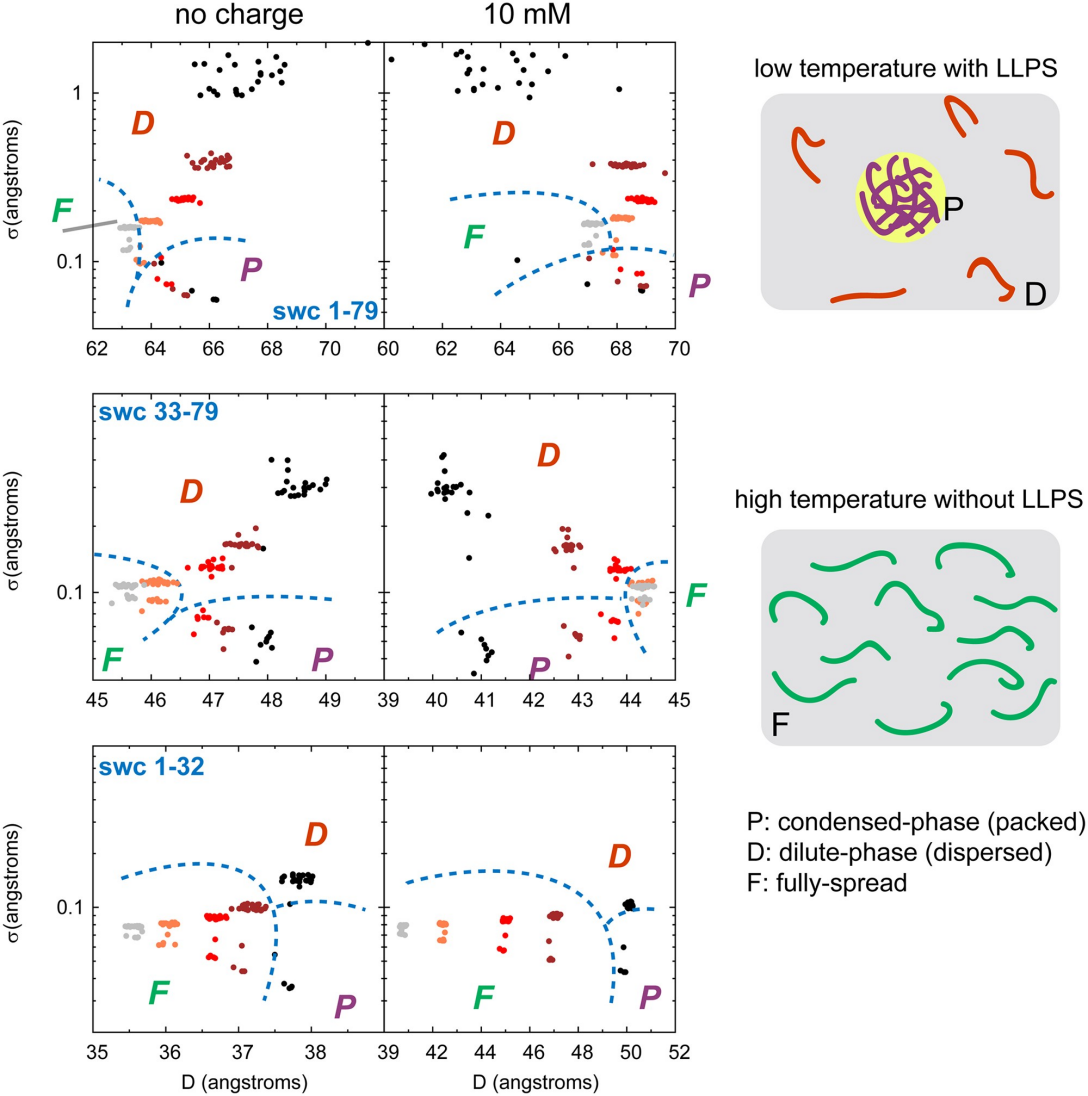
Predicted phase diagrams. Phase diagrams of each system with the distribution of *D* and *σ* (left), as well as the schematic diagrams for three forms of swc chains (right). The data in the left panels are extracted from the Fig 6, with the same coloring method. Three forms of swc chains (P, D, and F) are labeled in this figure. P represents the packed form of IDP in the condensed-phase (droplet) at low temperatures; D represents the dispersed form of IDP in the dilute-phase (outside the droplet) at low temperatures; F represents the fully-spread form of IDP at high temperatures without LLPS.

As shown in Fig 6, at low temperatures with LLPS, the swc chains are more flexible in the dilute-phase (high |*z*|) than in the condensed-phase (low |*z*|). For example, at 1.0 *T*_0_, all the *σ* values of swc 1–79 chains outside the condensed-phase (|*z*|> 200 Å) are higher than 0.9 Å (with and without charge interactions), while almost all the *σ* values of swc 1–79 chains inside the condensed-phase (|*z*|< 200 Å) are lower than 0.1 Å (with and without charge interactions, see Fig 7). At high temperatures without LLPS (4.0 *T*_0_), all swc 1–79 chains have similar but slightly higher *σ* value (about 0.2 Å) as those in the condensed phase at 1.0 *T*_0_ (with and without charge interactions). In addition, the distribution of the *D* values of swc 1–79 is more concentrated at 4.0 *T*_0_ than those at lower temperatures. And the mean *D* value of swc 1–79 at 4.0 *T*_0_ is lower than those at lower temperatures (see Fig 5B and 5C). The swc 33–79 and swc 1–32 have similar trends of the *σ* values along the |*z*|. These results suggest that the ensemble of swc chains exhibit different conformations with and without LLPS. When LLPS occurs, the lower *σ* values in the condensed-phase mean that swc chains are tightly packed, and the higher *σ* values in the dilute-phase mean that swc chains flexibly moving. At high temperatures without LLPS, swc chains are fully spread/distributed in the simulation box with relatively stable *D* and small *σ* values, which suggests that the high temperatures can decrease the flexibility of swc chains compared with those in the dilute-phase at low temperatures. As a result, we can classify the chains into three main forms (see Fig 7): packed form (P) in the condensed-phase with low *σ* value (low temperature with LLPS); dispersed form (D) in the dilute-phase with high *σ* value (low temperature with LLPS); fully-spread form (F) with low *σ* value (high temperature without obvious LLPS). Moreover, these three forms of swc chains can be distinguished on the phase diagram of *D* and *σ* (see the left panels of Fig 7). At low temperatures, swc chains behave in the dilute-phase as free IDPs/IDRs because there are almost no inter-chain interactions and the *D* value is flexible (high *σ*). The *D* value is stable in the condensed-phase because of the packing of the chains. P form (ordered behavior) and D form (disordered behavior) of swc chains can coexist at low temperatures. As the increase of the temperature, the boundary between the condensed-phase and the dilute-phase disappears (no LLPS). The simulation box is fully filled with swc chains. However, the thermodynamical motions are enhanced at high temperatures. The swc chains have to be rolled up a bit to avoid colliding with each other, leading to a lower *D* value at high temperatures.

The three forms of swc chains (P, D, and F) can also be recognized in the distribution of *E*_*inter*_ and *E*_*intra*_. As shown in Supporting information S12 and S13 Figs, at the low temperatures when LLPS occurs, chains in the packed form (P) have much higher *E*_*inter*_ and lower *E*_*intra*_ than those in the dispersed form (D). In addition, the standard error values of *E*_*intra*_ in D form (higher than 0.6 for swc 1–79 at 1.0 *T*_0_) are significantly higher than those in the P form (lower than 0.07 for swc 1–79 at 1.0 *T*_0_), which indicates that swc chains behave as free IDPs/IDRs in the dilute-phase. At high temperatures without LLPS, swc chains behave as the fully-spread form (F) with a bit higher *E*_*inter*_ (about 0.3 for swc 1–79 at 4.0 *T*_0_) than those in D form at low temperatures (close to 0 for swc 1–79 at 1.0 *T*_0_). In addition, the *E*_*intra*_ values of F form at high temperatures (ranging from 12.4 to 12.8 for swc 1–79 at 4.0 *T*_0_) show large differences with that of D form at low temperatures (ranging from 14.2 to 17.5 for swc 1-79 at 1.0 *T*_0_). The results suggest that at high temperature without LLPS, the chains in F form do not behave as free IDPs/IDRs.

The charge interactions have different impacts on the three swc segments. In the condensed-phase (packed form) at low temperatures, swc 1–79 and swc 1–32 are more expanded (larger *D*) at 10 mM than that without charge interactions, while swc 33–79 is more folded (smaller *D*) at 10 mM (see Fig 6). The results suggest that the more expanded conformation at 10 mM does not favor the stable packing in the condensed-phase because the *T*_*cr*_ values of both swc 1–79 and swc 1–32 are decreased at 10 mM. In addition, the large number of continuous negative-charged residues in swc 1–79 and swc 1–32 (the DEF/Y motifs) can form high inter-chain repulsive interactions. The high strength of electrostatic interactions (at low salt concentration) may not be helpful for the LLPS.

Intriguingly, in some systems with high extent of LLPS (e.g. swc 1–79 and 33–79 at 1.0 *T*_0_), it is obvious that the *D* value decreases slightly in the dilute-phase compared to that in the condensed-phase at 10 mM salt concentration, but increases slightly without charge interactions when LLPS occurred (see Fig 6). At 1.0 *T*_0_ without the charge interactions, the mean *D* of swc 1–79 is 66.00 Å in the condensed-phase (|*z*|< 200 Å) and 67.25 Å in the dilute-phase (|*z*|> 200 Å). At 10 mM, the mean *D* of swc 1–79 is 68.37 Å in the condensed-phase (|*z*|< 200 Å) and 64.03 Å in the dilute-phase (|*z*|> 200 Å). For swc 33–79, these values are 47.94 Å (condensed-phase, |*z*|< 400 Å) and 48.53 Å (dilute-phase, |*z*|> 400 Å) without charge interactions, 41.01 Å (condensed-phase, |*z*|< 400 Å) and 40.37 Å (dilute-phase, |*z*|> 400 Å) at 10 mM. As a result, the large amount of inter-chain interactions limit the proteins in the condensed-phase into a different form (P form), which is stable and different from the free IDP conformation (D form), keeping the shape of the droplet (MLO) stable. When LLPS is obvious, it seems that the vdW inter-chain interactions (no charges) will lead to a lower *D* in the condensed-phase than that in the dilute-phase. Combined vdW and charged inter-chain interactions (10 mM) will lead to a higher *D* in the condensed-phase than that in the dilute-phase. In addition, the fluctuation of the condensed-phase and the dilute-phase increases and decreases as the increase of the temperature. The difference between the condensed-phase and the dilute-phase becomes smaller until both of them transfer to the F form (LLPS disappears). And these behaviors above are found in the swc chains with different sequence length and charge pattern.

## Discussion

In this study, we measure the phase diagram of the three swc chains and show the influence of electrostatic interactions on the LLPS of these swc chains. We find that without electrostatic interactions, the three swc chains all show LLPS at low temperature (1.0 *T*_0_, see Fig 2). It is consistent with the theoretical conclusions of Dignon and Das *et al*. [27, 31]. The simulations of FUS did not show the ionic strength dependence [31]. Das *et al*. claimed that the general inter-residue LJ attraction favors LLPS but diminishes sequence specificity of LLPS [27]. We obtain similar conclusion because these proteins are neutral polymers with identical beads with no electrostatic interactions. The extent of LLPS is only relevant with the sequence length.

In addition, our simulation results suggest that the electrostatic interactions can increase the critical temperature *T*_*cr*_ of swc 33–79 a bit, but decrease the *T*_*cr*_ of swc 1–79 and 1–32 (Fig 2). Intriguingly, it is different from the simulation results of Das *et al*. that *T*_*cr*_ values of sv1 and sv15 all decrease when the electrostatic interactions are screened [27]. In that study, both sv1 and sv15 are ideal polyampholytes with equal number of positive-charged residues and negative-charged residues (*f*_+_ = *f*_−_ = 0.5, *f*_+_ and *f*_−_ are the fractions of positive-charged and negative-charged residues, respectively). In the present study, the conserved DEF/Y-1 and DEF/Y-2 motifs enhance the *f*_−_ values of swc 1–79 and swc 1–32 significantly (see Fig 1). Both swc 1–79 and 1–32 have high NCPR (net charge per residue, |*f*_+_ − *f*_−_|) that can be considered as polyelectrolytes according to the classification of Das and Pappu [35]. We then performed the simulation using the sv1 and sv15 sequences with the same settings. As shown in the Supporting information S14 Fig, the *T*_*cr*_ of sv1 seems barely changed when the electrostatic interactions are strong or screened. The *T*_*cr*_ of sv15 is about 3.5 *T*_0_ at 10 mM, and decreases to about 3.0 *T*_0_ when the electrostatic interactions are screened. And the electrostatic interactions influence the *P*_*H*_ − *P*_*L*_ of sv15 significantly at low temperatures. We obtain the same result with Das *et al*. that the *T*_*cr*_ of sv15 is higher than that of sv1. The differences on the value and magnitude could result from the different simulation model and box. The SCD values of both sv1 and sv15 are negative, -0.41 and -4.35. In addition, the SCD values of swc 1–32, swc 33–79, and swc 1–79 are 7.77, -0.32, and 7.40. Combined with the results of swc sequences, we can conclude that for the sequence with high negative SCD value (e.g. sv15 with -4.35), the electrostatic interactions will favor the LLPS. On the other hand, for the sequence with high positive SCD value (e.g. swc 1–32 with 7.77), the electrostatic interactions will hamper the LLPS. Moreover, though swc 33–79 and sv1 have similar sequence lengths and SCD values, the number and distribution of their charged residues are all different (sv1 and sv15 are fully charged polyampholytes with zero net charge), which may lead to different density distribution and LLPS behaviors.

Some experiments have demonstrated that the arginine-rich positively charged sequences (e.g., RGG boxes, RRMs, SR repeats) drive the LLPS in an ionic strength dependent manner of Ddx4, LAF-1, and hnRNPA1 [2, 6, 7, 15, 45]. Besides, both methylation and phosphorylation can change the phase separation threshold for certain proteins such as Ddx4, tau441, MEG-3/MEG-4 [2, 45, 46]. Therefore, the LLPS behavior in different solutions/environments will be more complicated *in vivo* or in the cell.

We have summarized the behavior of swc ensemble under the influence of temperature and found a different phenomenon from the conventional free IDPs/IDRs. Swc chains tend to be a bit more expanded at lower temperatures when there are no charge interactions. In the swc ensemble, the chains behave as two forms coexisted with different *D* and *σ* values at low temperatures, and have to be rolled up a bit to avoid colliding with each other at high temperatures, leading to a lower *D* value compared with the extended structure. In the previous studies [19, 47, 48], the discovery of IDPs has extended the traditional sequence-structure relationship and led to the protein trinity model, which suggests that the native protein structure includes not only the ordered state (solid-like), but also molten globules (MG, liquid-like) and the random coils (pre-MG, gas-like). Free IDPs are found to behave like molten globules (collapsed-disordered) or random coils (extended-disordered). However, the trinity property is suitable for the single IDP or the IDPs without inter-chain interactions between each other (free IDPs). Here in this study, we discussed the structure and order of the whole ensemble of swc chains. Three different forms of swc chains can be distinguished on the phase diagrams of *D* and *σ* values (see Fig 7): packed form (P) in the condensed-phase with low *σ* value (low temperature with LLPS); dispersed form (D) in the dilute-phase with high *σ* value (low temperature with LLPS); fully-spread form (F) with low *σ* value (high temperature without obvious LLPS). The swc conformations can be linked with the temperature and the ensemble. P and D forms can coexist at low temperatures. They disappear into another form (F) at high temperatures. In addition, some characteristics, such as the *D* and *σ* values inside and outside the droplet, are found in the swc chains with different sequence lengths and charge patterns. It suggests that similar behaviors may be observed in other IDPs/IDRs. Different structures can be linked to different protein functions. Likewise, different forms of IDP/IDR ensemble may facilitate the essential processes in cell.

There are significant investigations that have discussed the biological functions of the LLPS. Huang *et al*. predicted that the IDRs of Swc5 may be involved in the LLPS with the similar mode as the carboxy-terminal domain (CTD) of RNA polymerase (Pol) II [33]. For Pol II, the CTD (IDR) can undergo LLPS *in vitro*, which is based mainly on the weak hydrophobic interactions and influenced by the CTD length and the CTD phosphorylation [49]. Boehning *et al*. [49] proposed that the CTD can recruit the core of Pol II and the transcription factors on the condensed-phase (droplet), leading to the Pol II hub, which may provide a reservoir of Pol II, enabling high initiation rates during the activated transcription. Moreover, as a histone chaperon, the Swc5 binds with H2A-H2B tightly with its IDR [33]. The DEF/Y motifs are essential for the binding. Therefore, the IDR of Swc5, especially the DEF/Y motifs, may recruit or retain histones within phase-separated liquid droplets where transcription factors and chromatin remodeling factors coalesce to regulate transcription [33]. Our investigations show the LLPS of the Swc5 IDR, which may facilitate the recruitment, deposit, and assembly of the histones. In addition, our results agree with the properties of the CTD about the LLPS, suggesting the similar assembly mode and biological function of the IDR of Swc5.

## Supporting Information

S1 Fig Parameters calibration. The mean head–end distance (D) with different parameters *σ* and *ε* (left panel: *σ* = 1.0 nm, *ε* = 0.01, 0.1, 0.8 kJ/mol; right panel: *σ* = 0.6, 0.8, 1.0 nm, *ε* = 0.01 kJ/mol).
(TIF)

S2 Fig The distribution of the peak1 of swc 1–79. Peak1 is the *r* value corresponding to the peak value of *g*(*r*) curve between chain 1 and another chain (199 peak1 values in total).
(TIF)

S3 Fig The distribution of the peak1 of swc 33–79. Peak1 is the *r* value corresponding to the peak value of *g*(*r*) curve between chain 1 and another chain (199 peak1 values in total).
(TIF)

S4 Fig The distribution of the peak1 of swc 1–32. Peak1 is the *r* value corresponding to the peak value of *g*(*r*) curve between chain 1 and another chain (199 peak1 values in total).
(TIF)

S5 Fig *P*_*H*_ − *P*_*L*_ of swc 1–32 as a function of simulation time. The upper panel shows the simulations without charge interactions (no charge); the bottom panel shows the simulations at 10 mM salt concentration.
(TIF)

S6 Fig *P*_*H*_ − *P*_*L*_ of swc 33–79 as a function of simulation time. The upper panel shows the simulations without charge interactions (no charge); the bottom panel shows the simulations at 10 mM salt concentration.
(TIF)

S7 Fig *P*_*H*_ − *P*_*L*_ of swc 1–79 as a function of simulation time. The upper panel shows the simulations without charge interactions (no charge); the bottom panel shows the simulations at 10 mM salt concentration.
(TIF)

S8 Fig Swc chain movements. The displacement of one swc chain to the CM of all 200 swc 1–79 chains on the *z* axis (displacement z) during the simulation. Considering the periodic simulation box, the displacement is the minimum of the distance values between this representative chain and any of the system CM mirrors.
(TIF)

S9 Fig Swc chain movements. The displacement of one swc chain to the CM of all 200 swc 33–79 chains on the *z* axis (displacement z) during the simulation. Considering the periodic simulation box, the displacement is the minimum of the distance values between this representative chain and any of the system CM mirrors.
(TIF)

S10 Fig Swc chain movements. The displacement of one swc chain to the CM of all 200 swc 1–32 chains on the *z* axis (displacement z) during the simulation. Considering the periodic simulation box, the displacement is the minimum of the distance values between this representative chain and any of the system CM mirrors.
(TIF)

S11 Fig The distribution of the probability (A) and the standard error (B) as a function of displacement |*z*|. The coloring method is the same as that in Fig 2.
(TIF)

S12 Fig Mean number of inter-chain (*E*_*inter*_) and intra-chain (*E*_*intra*_) electrostatic attractions of swc 1–79 (A), swc 33–79 (B), and swc 1–32 (C) as a function of |*z*|. Average and standard error values during the last 1000 *τ* simulation data are illustrated in this figure.
(TIF)

S13 Fig Phase diagrams of each system with the distribution of *E*_*inter*_ and *E*_*intra*_. The data points are extracted from the figure above, with the same coloring method. Three forms of swc chains (P, D, and F) are labeled in this figure. P represents the packed form of IDP in the condensed-phase (droplet) at low temperatures; D represents the dispersed form of IDP in the dilute-phase (outside the droplet) at low temperatures; F represents the fully-spread form of IDP at high temperatures without LLPS.
(TIF)

S14 Fig The appearance and disappearance of phase separation. (A) Phase diagram of sv1 and sv15 in different solvents. (B) *P*_*H*_ − *P*_*L*_ changes with temperature and solvent. Here *P*_*H*_ and *P*_*L*_ are the highest and the lowest points of sv residue distribution along the z axis.
(TIF)

S1 Table The *P*_*H*_ − *P*_*L*_ data of different sequences at different condition (temperature 1.0, 1.5, 2.0, 3.0, 4.0 *T*_0_; 10 mM salt concentration (10) or without charge interactions (no)). These data are the mean values calculated with the last 1000 *τ* trajectory.
(DOCX)

S2 Table The head–end data of different sequences at different condition (temperature 1.0, 1.5, 2.0, 3.0, 4.0 *T*_0_; at 10 mM salt concentration (10) or without charge interactions (no)). These data are the mean values calculated with the last 1000 *τ* trajectory.
(DOCX)
