# Supplementary figures and images for: Thermodynamic and sequential characteristics of phase separation and droplet formation for an intrinsically disordered region/protein ensemble
Source: PLoS Comput Biol. 2021 Mar 8;17(3):e1008672. doi: 10.1371/journal.pcbi.1008672 (PMC7939360; doi:10.1371/journal.pcbi.1008672)

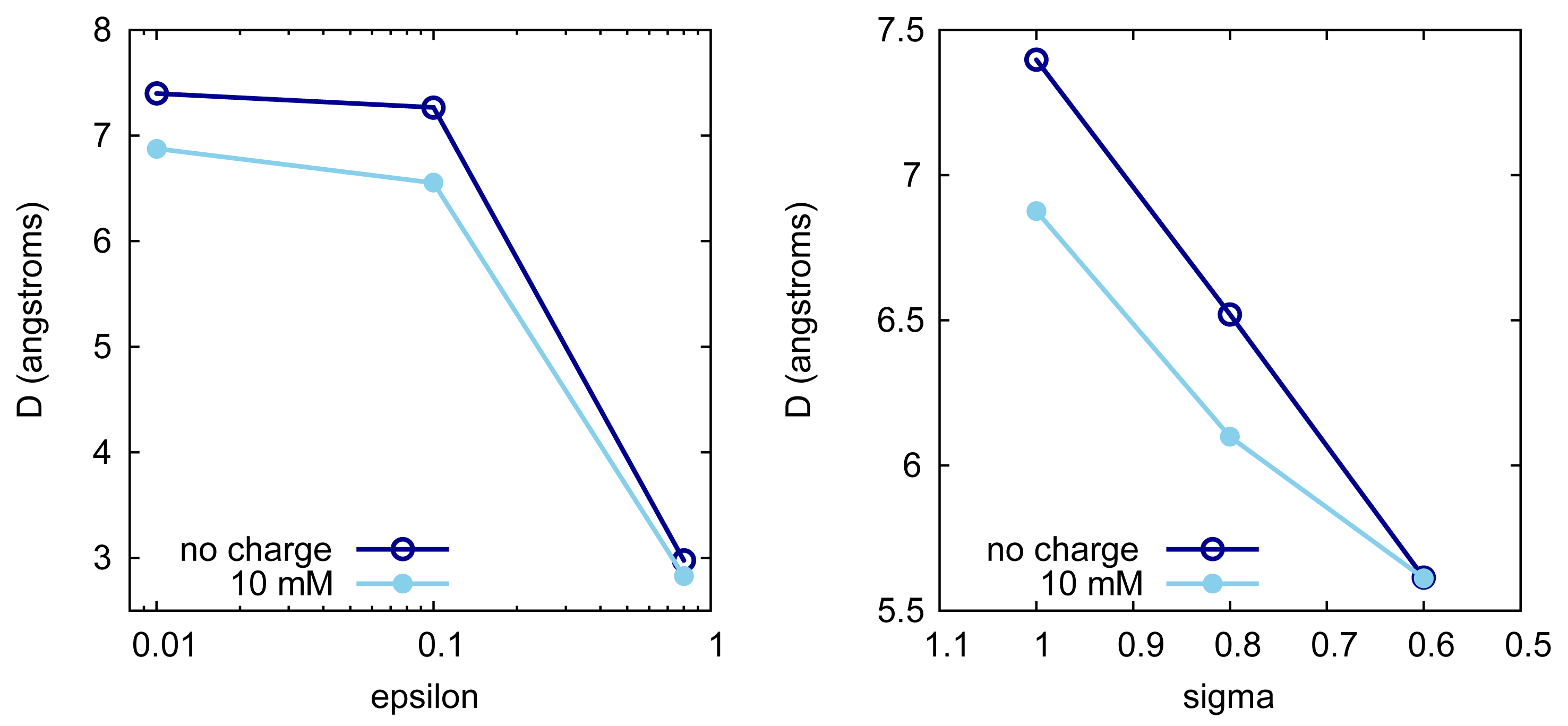

Supplement: S1 Fig — The mean head–end distance (D) with different parameters σ and ε (left panel: σ = 1.0 nm, ε = 0.01, 0.1, 0.8 kJ/mol; right panel: σ = 0.6, 0.8, 1.0 nm, ε = 0.01 kJ/mol). (TIF) [file pcbi.1008672.s001.tif]

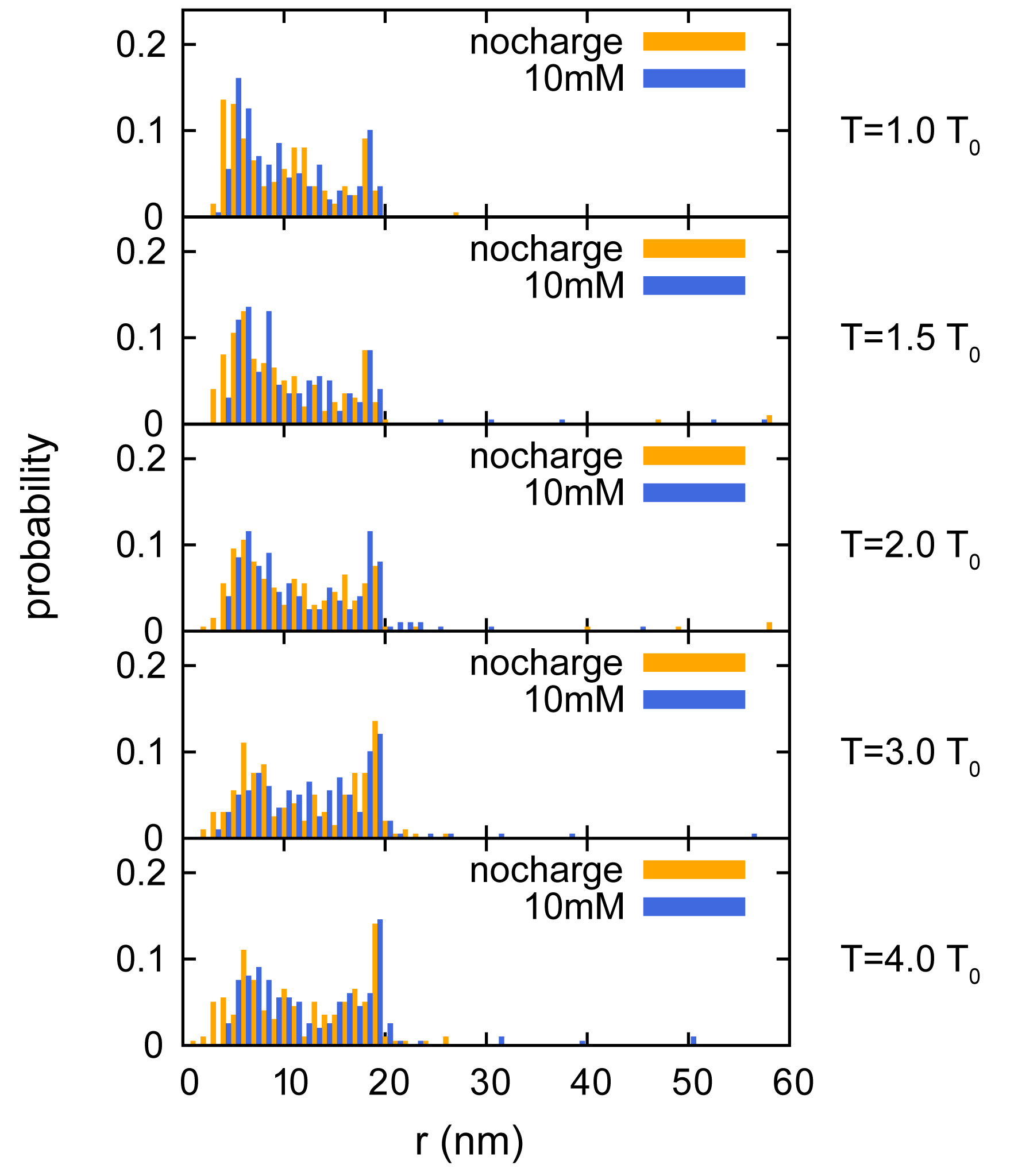

Supplement: S2 Fig — Peak1 is the r value corresponding to the peak value of g(r) curve between chain 1 and another chain (199 peak1 values in total). (TIF) [file pcbi.1008672.s002.tif]

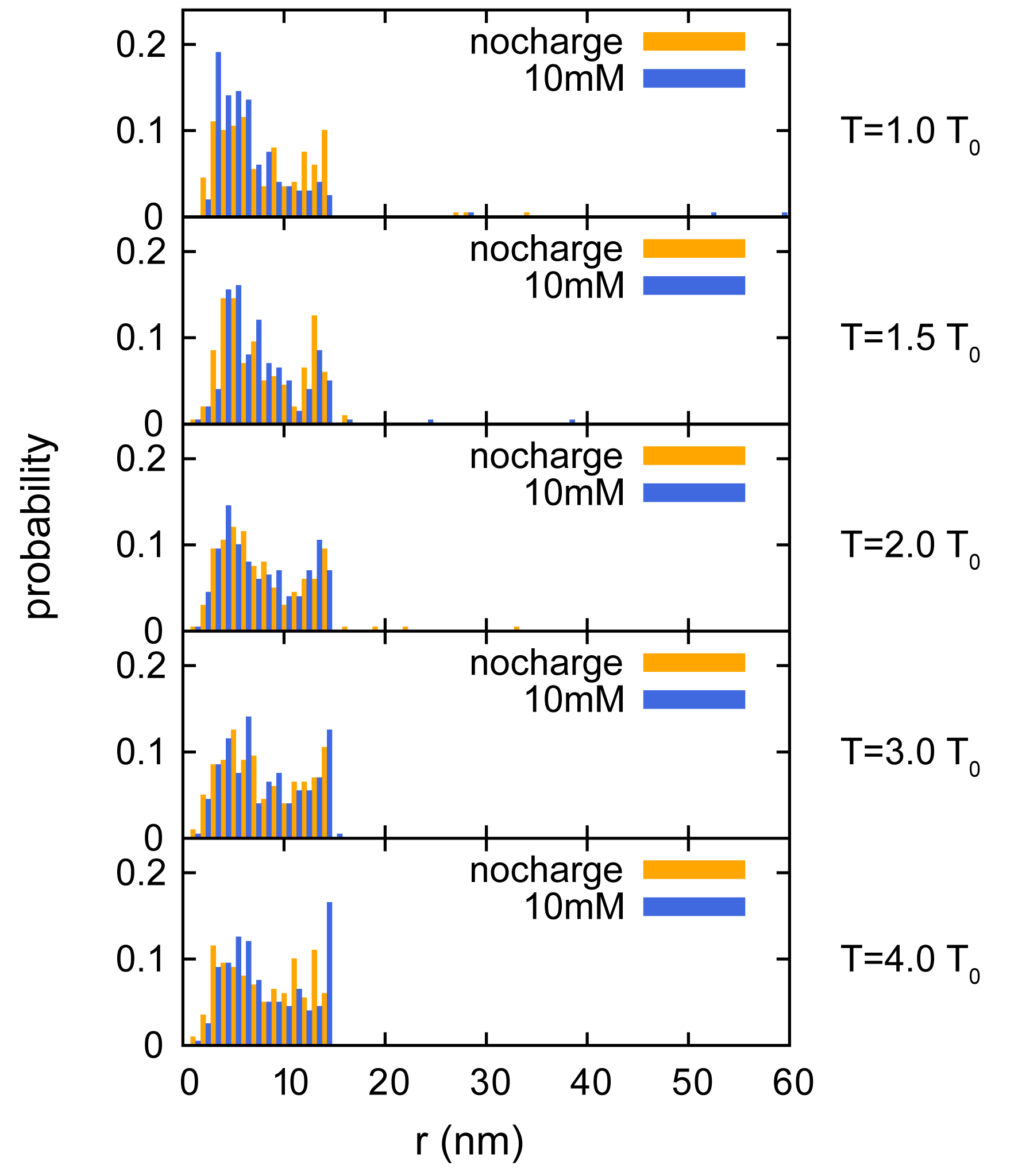

Supplement: S3 Fig — Peak1 is the r value corresponding to the peak value of g(r) curve between chain 1 and another chain (199 peak1 values in total). (TIF) [file pcbi.1008672.s003.tif]

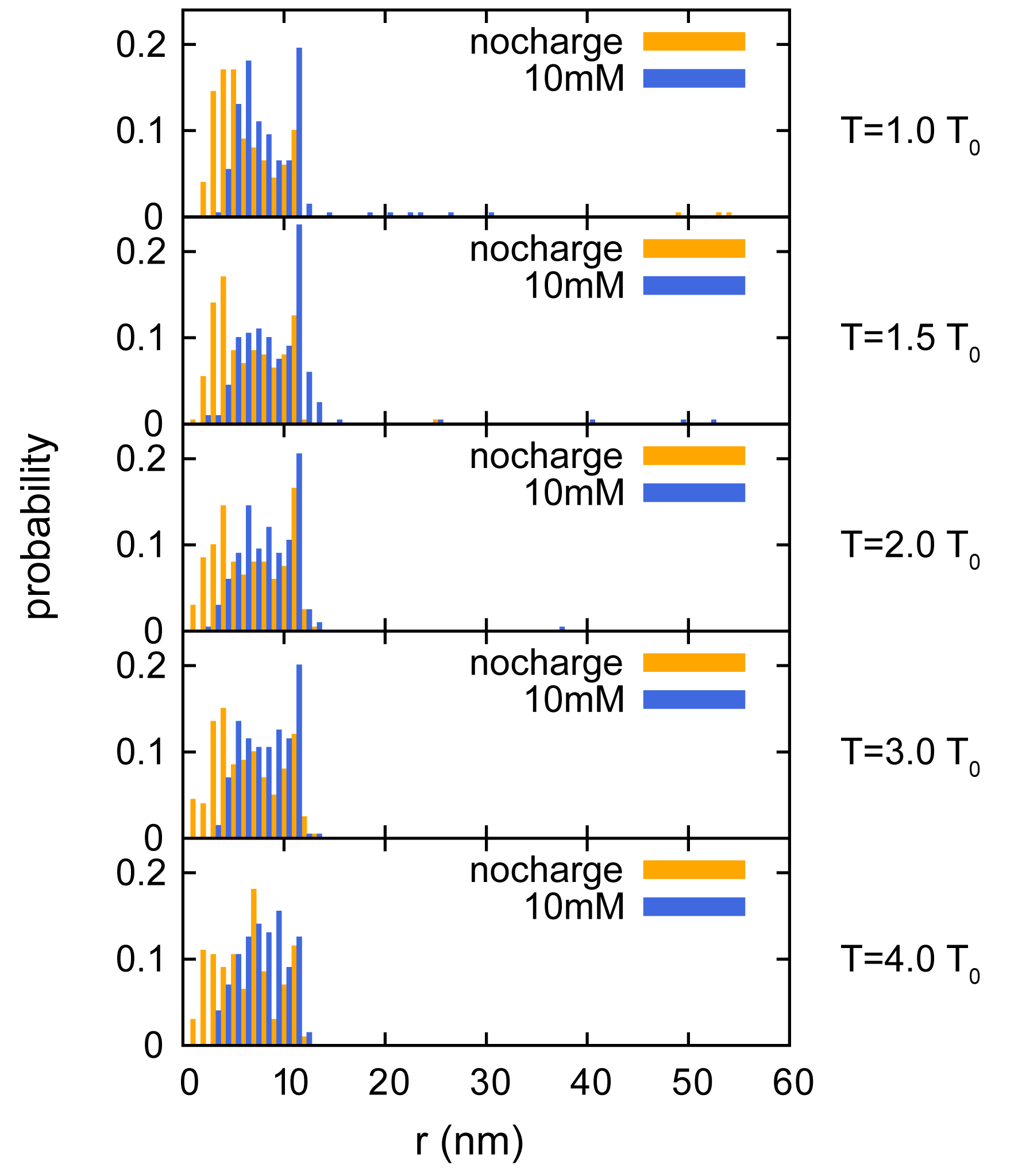

Supplement: S4 Fig — Peak1 is the r value corresponding to the peak value of g(r) curve between chain 1 and another chain (199 peak1 values in total). (TIF) [file pcbi.1008672.s004.tif]

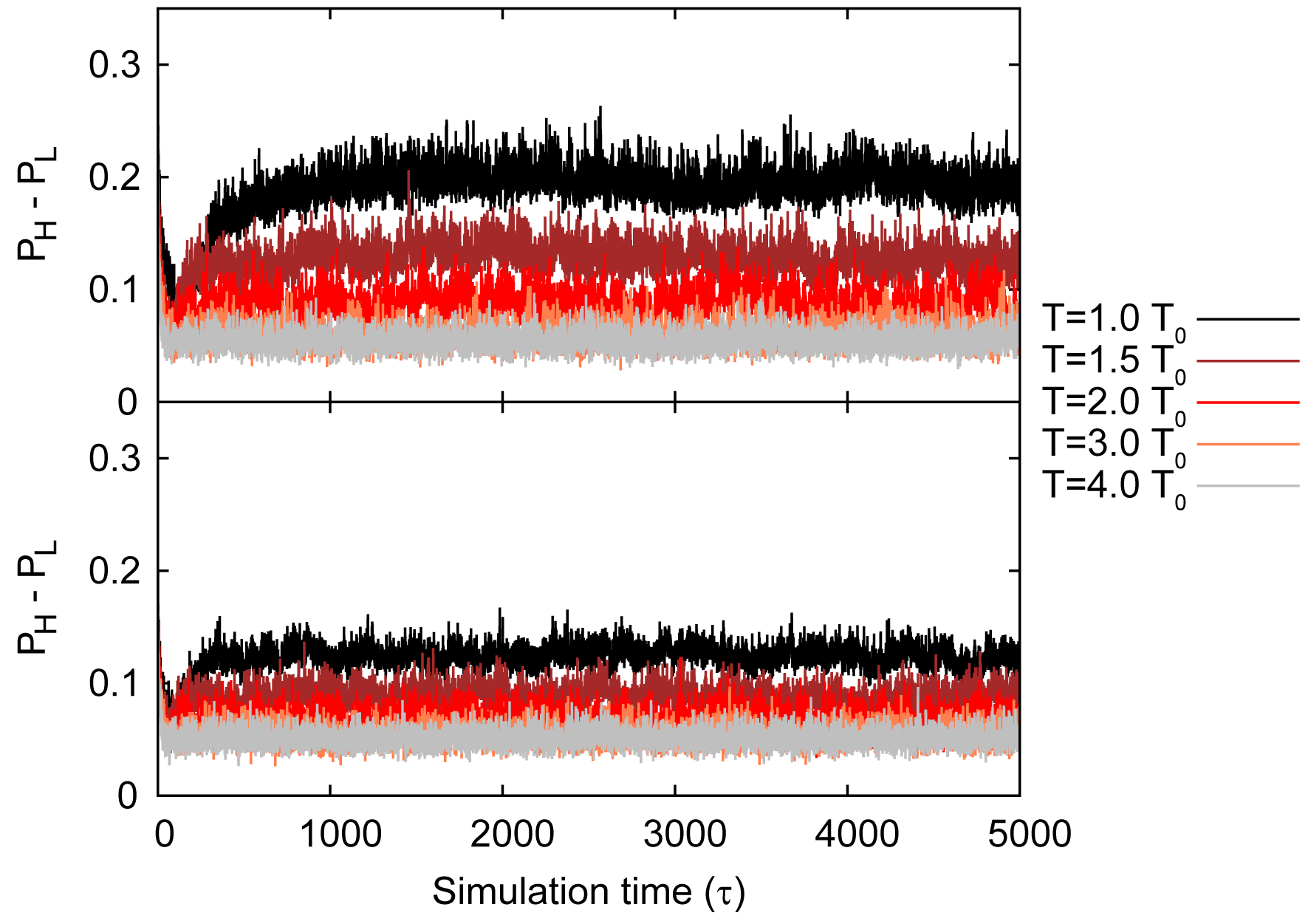

Supplement: S5 Fig — The upper panel shows the simulations without charge interactions (no charge); the bottom panel shows the simulations at 10 mM salt concentration. (TIF) [file pcbi.1008672.s005.tif]

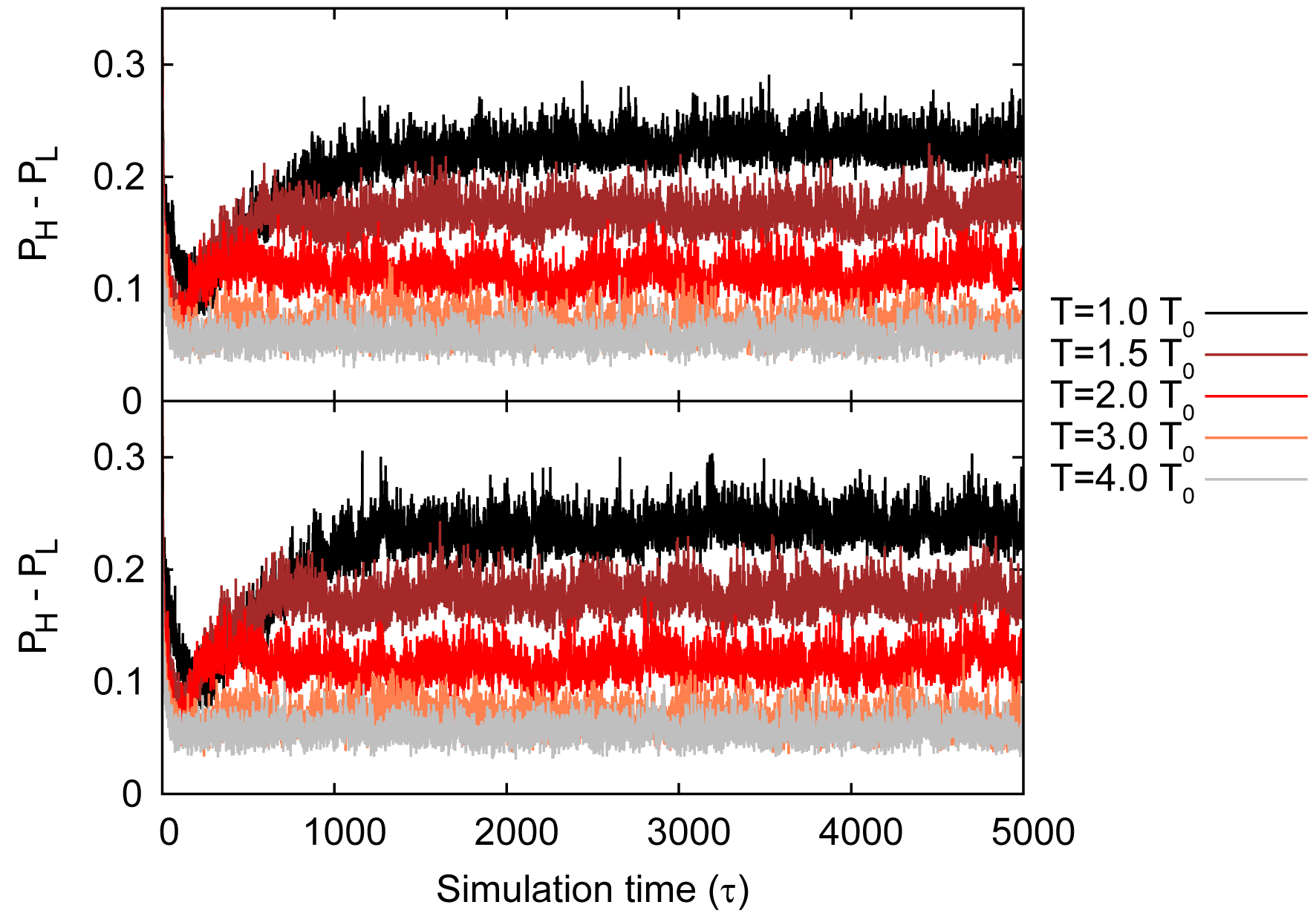

Supplement: S6 Fig — The upper panel shows the simulations without charge interactions (no charge); the bottom panel shows the simulations at 10 mM salt concentration. (TIF) [file pcbi.1008672.s006.tif]

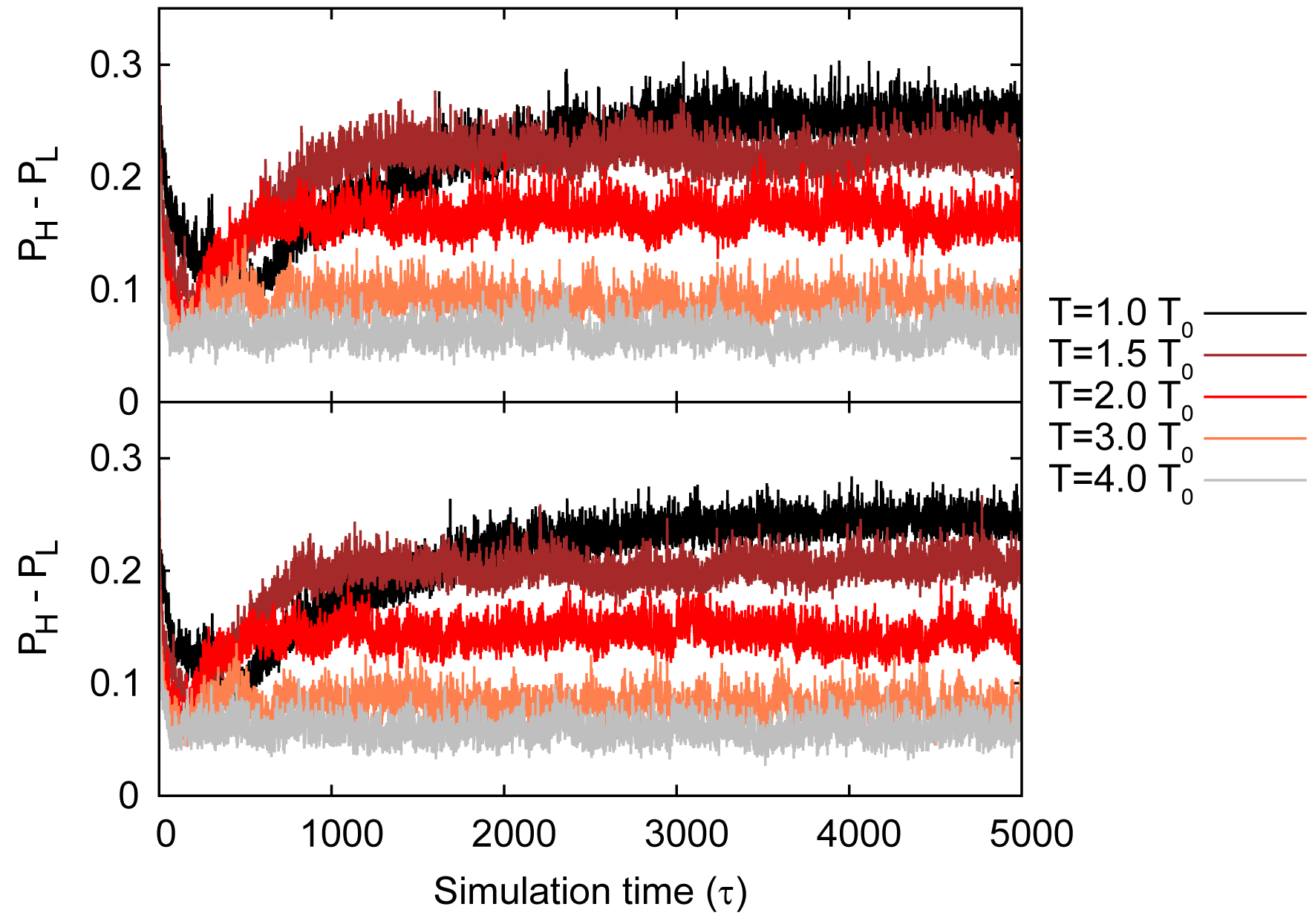

Supplement: S7 Fig — The upper panel shows the simulations without charge interactions (no charge); the bottom panel shows the simulations at 10 mM salt concentration. (TIF) [file pcbi.1008672.s007.tif]

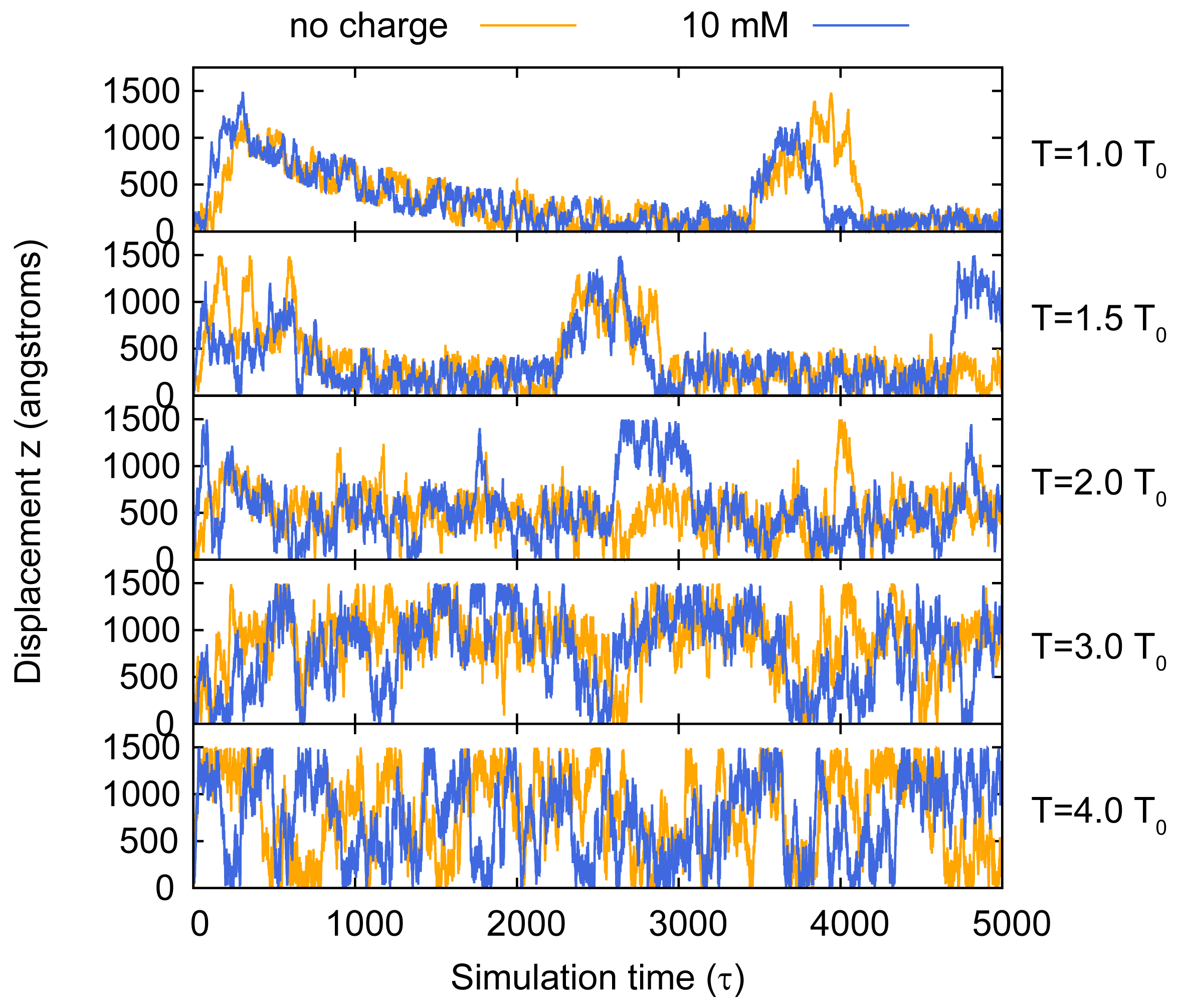

Supplement: S8 Fig — The displacement of one swc chain to the CM of all 200 swc 1–79 chains on the z axis (displacement z) during the simulation. Considering the periodic simulation box, the displacement is the minimum of the distance values between this representative chain and any of the system CM mirrors. (TIF) [file pcbi.1008672.s008.tif]

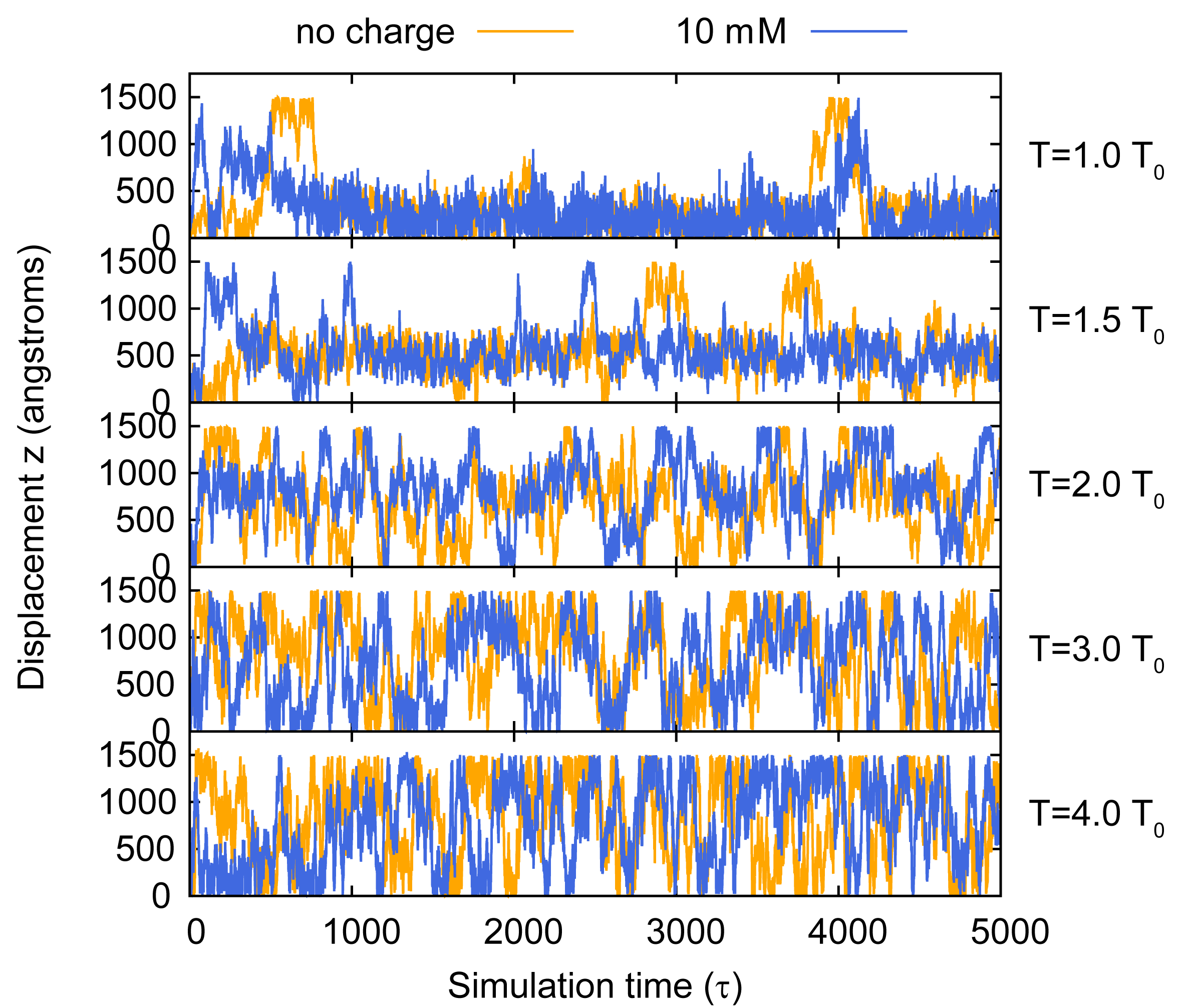

Supplement: S9 Fig — The displacement of one swc chain to the CM of all 200 swc 33–79 chains on the z axis (displacement z) during the simulation. Considering the periodic simulation box, the displacement is the minimum of the distance values between this representative chain and any of the system CM mirrors. (TIF) [file pcbi.1008672.s009.tif]

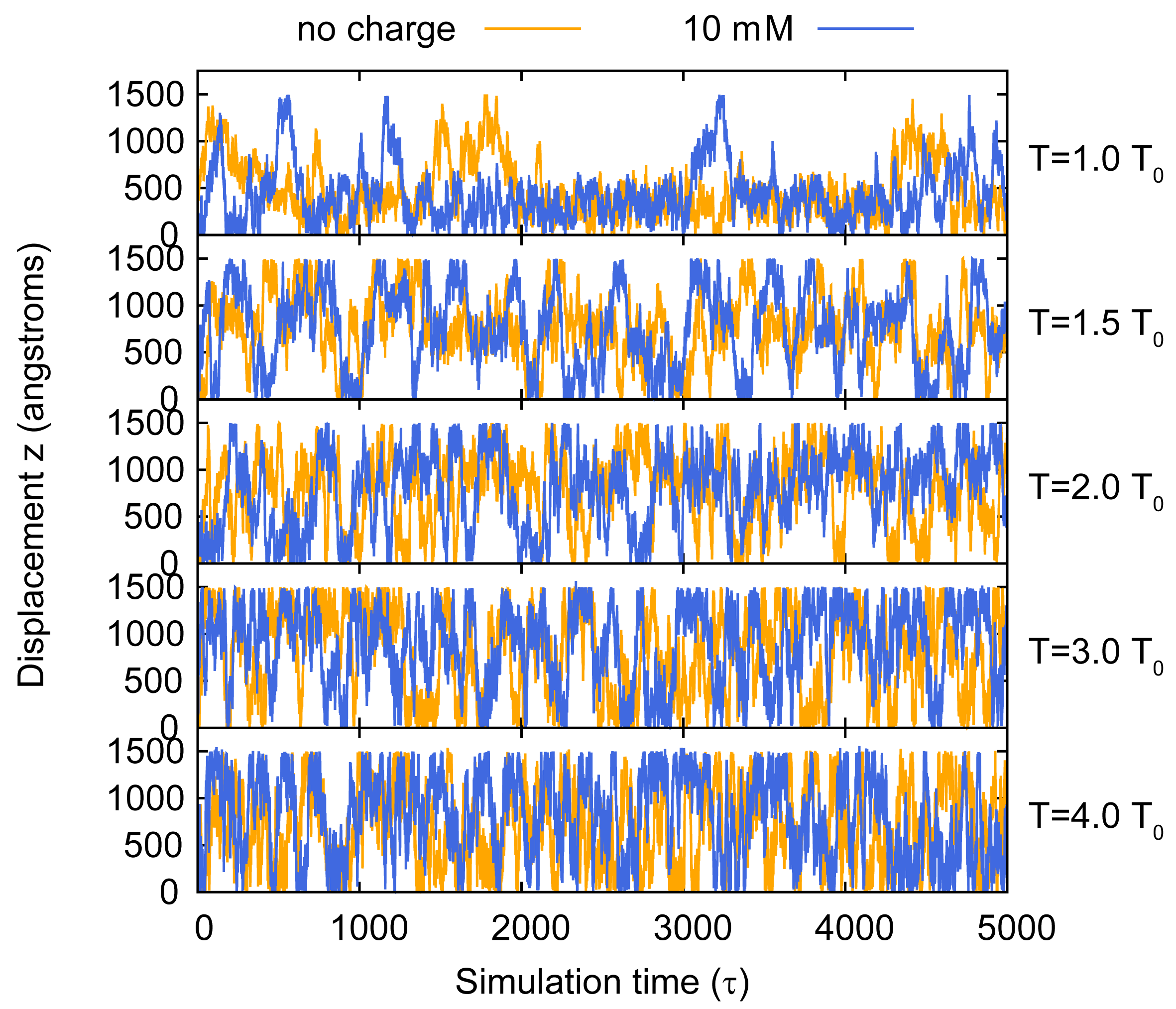

Supplement: S10 Fig — The displacement of one swc chain to the CM of all 200 swc 1–32 chains on the z axis (displacement z) during the simulation. Considering the periodic simulation box, the displacement is the minimum of the distance values between this representative chain and any of the system CM mirrors. (TIF) [file pcbi.1008672.s010.tif]

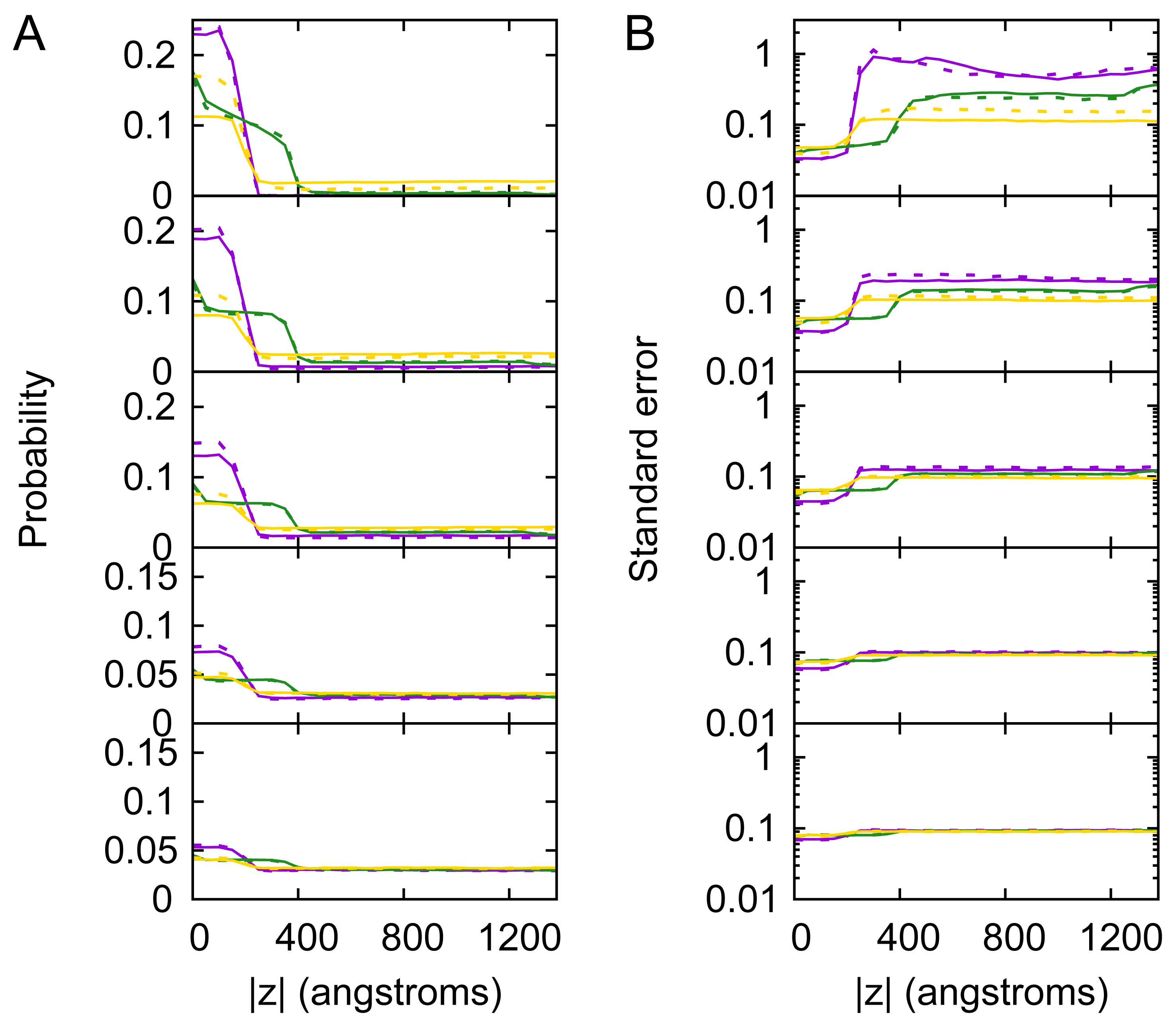

Supplement: S11 Fig — The coloring method is the same as that in Fig 2. (TIF) [file pcbi.1008672.s011.tif]

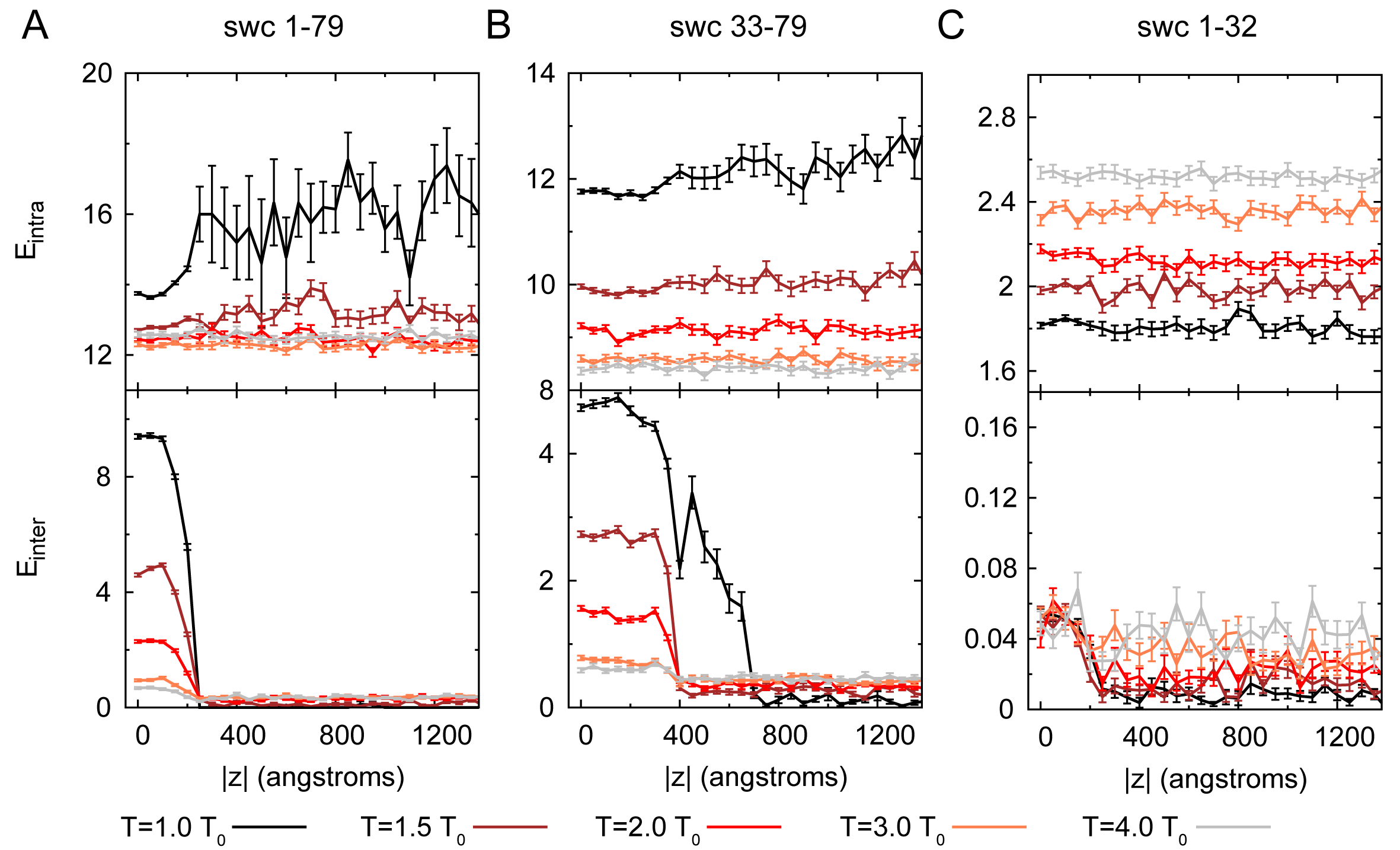

Supplement: S12 Fig — Average and standard error values during the last 1000 τ simulation data are illustrated in this figure. (TIF) [file pcbi.1008672.s012.tif]

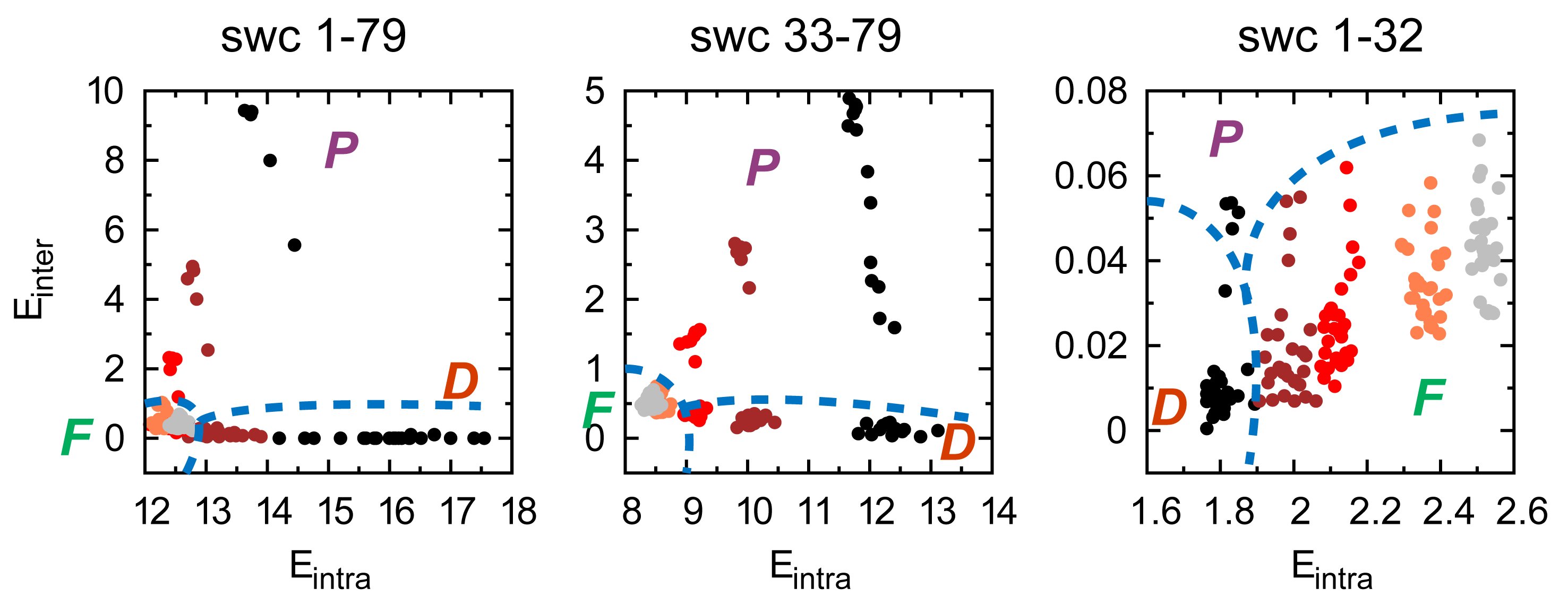

Supplement: S13 Fig — The data points are extracted from the figure above, with the same coloring method. Three forms of swc chains (P, D, and F) are labeled in this figure. P represents the packed form of IDP in the condensed-phase (droplet) at low temperatures; D represents the dispersed form of IDP in the dilute-phase (outside the droplet) at low temperatures; F represents the fully-spread form of IDP at high temperatures without LLPS. (TIF) [file pcbi.1008672.s013.tif]

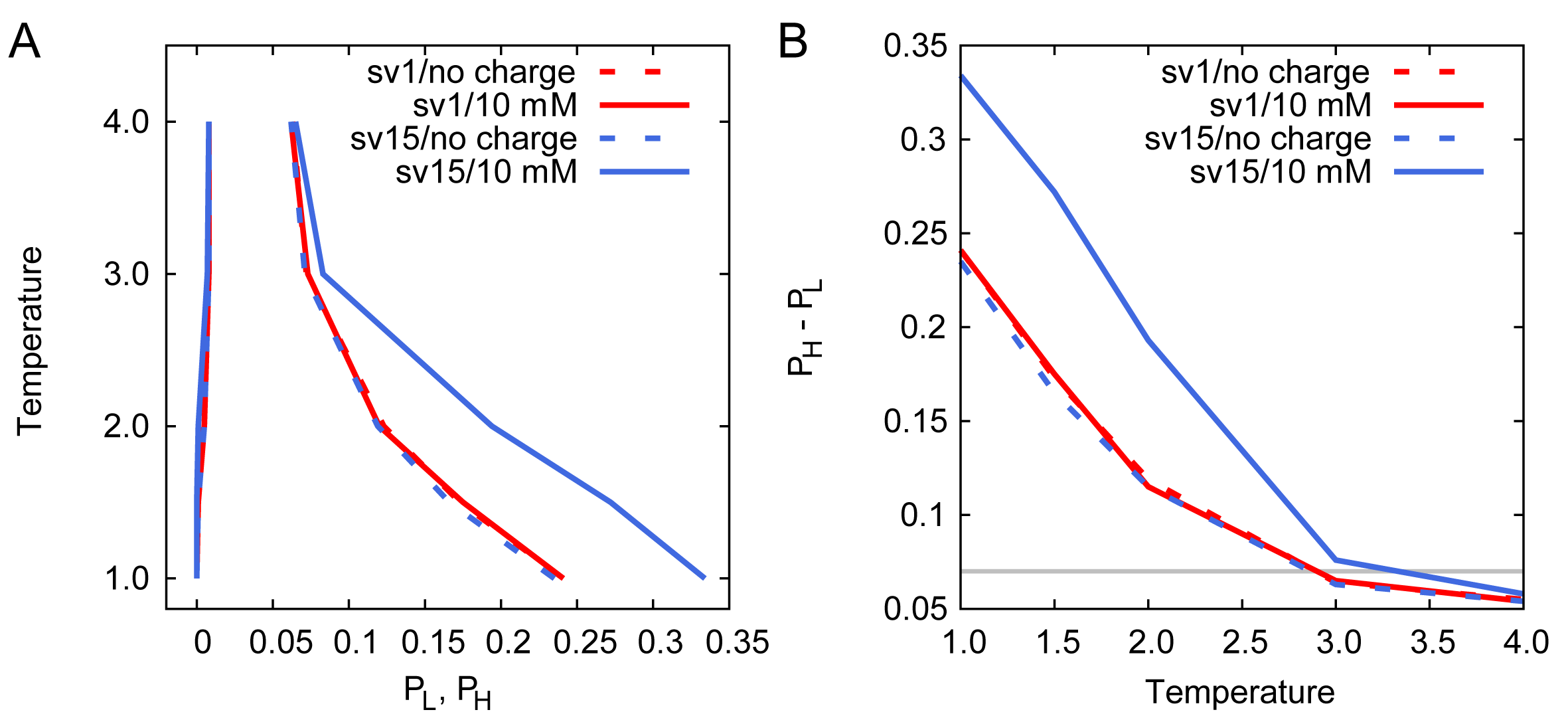

Supplement: S14 Fig — (A) Phase diagram of sv1 and sv15 in different solvents. (B) PH − PL changes with temperature and solvent. Here PH and PL are the highest and the lowest points of sv residue distribution along the z axis. (TIF) [file pcbi.1008672.s014.tif]
