# Supplementary material for: Thermodynamic and sequential characteristics of phase separation and droplet formation for an intrinsically disordered region/protein ensemble
Source: PLoS Comput Biol. 2021 Mar 8;17(3):e1008672. doi: 10.1371/journal.pcbi.1008672 (PMC7939360; doi:10.1371/journal.pcbi.1008672)
Supplement: S1 Table — These data are the mean values calculated with the last 1000 τ trajectory. (DOCX) [file pcbi.1008672.s015.docx]

S1 Table. The P_H_-P_L_ data of different sequences at different condition (temperature 1.0, 1.5, 2.0, 3.0, and 4.0 T_0_; 10 mM salt concentration (10) or without charge interactions (no)). These data are the mean values calculated with the last 1000 τ trajectory.

| system | temperature  solvent | 1.0 | 1.5 | 2.0 | 3.0 | 4.0 |
| --- | --- | --- | --- | --- | --- | --- |
| swc 1-79 | 10 | 0.244 | 0.207 | 0.146 | 0.078 | 0.061 |
|  | no | 0.251 | 0.223 | 0.160 | 0.086 | 0.065 |
| swc 33-79 | 10 | 0.239 | 0.178 | 0.121 | 0.064 | 0.056 |
|  | no | 0.228 | 0.171 | 0.117 | 0.063 | 0.054 |
| swc 1-32 | 10 | 0.121 | 0.089 | 0.068 | 0.054 | 0.051 |
|  | no | 0.191 | 0.124 | 0.087 | 0.061 | 0.055 |
| sv1 | 10 | 0.241 | 0.175 | 0.115 | 0.065 | 0.054 |
|  | no | 0.241 | 0.173 | 0.119 | 0.064 | 0.055 |
| sv15 | 10 | 0.334 | 0.272 | 0.193 | 0.076 | 0.058 |
|  | no | 0.235 | 0.165 | 0.115 | 0.063 | 0.054 |
