# Supplementary material for: Thermodynamic and sequential characteristics of phase separation and droplet formation for an intrinsically disordered region/protein ensemble
Source: PLoS Comput Biol. 2021 Mar 8;17(3):e1008672. doi: 10.1371/journal.pcbi.1008672 (PMC7939360; doi:10.1371/journal.pcbi.1008672)
Supplement: S2 Table — These data are the mean values calculated with the last 1000 τ trajectory. (DOCX) [file pcbi.1008672.s016.docx]

S2 Table. The head-end data of different sequences at different condition (temperature 1.0, 1.5, 2.0, 3.0, and 4.0 T_0_; 10 mM salt concentration (10) or without charge interactions (no)). These data are the mean values calculated with the last 1000 τ trajectory.

| system | temperature  solvent | 1.0 | 1.5 | 2.0 | 3.0 | 4.0 |
| --- | --- | --- | --- | --- | --- | --- |
| swc 1-79 | 10 | 67.979 | 68.456 | 68.685 | 68.072 | 67.102 |
|  | no | 65.953 | 65.171 | 64.708 | 63.925 | 63.240 |
| swc 33-79 | 10 | 40.950 | 42.892 | 43.771 | 44.333 | 44.354 |
|  | no | 47.968 | 47.359 | 46.905 | 46.063 | 45.577 |
| swc 1-32 | 10 | 50.010 | 46.942 | 44.998 | 42.405 | 40.743 |
|  | no | 37.740 | 37.133 | 36.636 | 36.032 | 35.579 |
| sv1 | 10 | 51.152 | 50.078 | 49.312 | 48.233 | 47.578 |
|  | no | 49.430 | 48.852 | 48.401 | 47.620 | 47.012 |
| sv15 | 10 | 29.554 | 30.754 | 30.036 | 33.496 | 37.4230 |
|  | no | 49.486 | 48.817 | 48.354 | 47.575 | 46.985 |
